# Morphologic, Phylogenetic and Chemical Characterization of a Brackish Colonial Picocyanobacterium (Coelosphaeriaceae) with Bioactive Properties

**DOI:** 10.3390/toxins8040108

**Published:** 2016-04-12

**Authors:** Kerstin Häggqvist, Anna Toruńska-Sitarz, Agata Błaszczyk, Hanna Mazur-Marzec, Jussi Meriluoto

**Affiliations:** 1 Biochemistry, Faculty of Science and Engineering, Åbo Akademi University, Artillerigatan 6A, Åbo 20520, Finland; jussi.meriluoto@abo.fi; 2 Department of Marine Biotechnology, Institute of Oceanography, University of Gdańsk, Al. Marszałka Piłsudskiego 46, Gdynia 81-378, Poland; anna.torunska@ug.edu.pl (A.T.-S.); agata.blaszczyk@ug.edu.pl (A.B.); biohm@ug.edu.pl (H.M.-M.)

**Keywords:** anabaenopeptins, cyanopeptolins, enzyme inhibition, polyphasic approach, *Snowella*, *Woronichinia*

## Abstract

Despite their cosmopolitan distribution, knowledge on cyanobacteria in the family Coelosphaeriaceae is limited. In this study, a single species culture of a coelosphaeran cyanobacterium isolated from a brackish rock pool in the Baltic Sea was established. The strain was characterized by morphological features, partial 16S rRNA sequence and nonribosomal oligopeptide profile. The bioactivity of fractionated extracts against several serine proteases, as well as protein-serine/threonine phosphatases was studied. Phylogenetic analyses of the strain suggested a close relationship with *Snowella litoralis*, but its morphology resembled *Woronichinia compacta*. The controversial morphologic and phylogenetic results demonstrated remaining uncertainties regarding species division in this cyanobacteria family. Chemical analyses of the strain indicated production of nonribosomal oligopeptides. In fractionated extracts, masses and ion fragmentation spectra of seven possible anabaenopeptins were identified. Additionally, fragmentation spectra of cyanopeptolin-like peptides were collected in several of the fractions. The nonribosomal oligopeptide profile adds another potential identification criterion in future inter- and intraspecies comparisons of coelosphaeran cyanobacteria. The fractionated extracts showed significant activity against carboxypeptidase A and trypsin. Inhibition of these important metabolic enzymes might have impacts at the ecosystem level in aquatic habitats with high cyanobacteria densities.

## 1. Introduction

Cyanobacteria are ample producers of secondary metabolites, *i.e.*, compounds not involved in primary metabolism. Among these metabolites, structurally diverse oligopeptides are regularly characterized. Some of the small peptides are synthesized on large nonribosomal peptide synthetase enzymes, composed of modules with several domains, each responsible for a specific function in the peptide formation. Each module generally incorporates one sequential amino acid in the peptide chain, but there are exceptions [1]. The structure of the peptide product depends on the arrangement of modules and domains, as well as on the substrate specificity of the activation domain.

**Figure 1:**
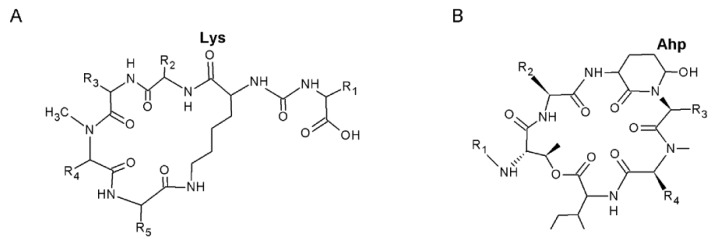
General structure of (**A**) anabaenopeptins, with the conserved Lys; and (**B**) cyanopeptolins, with the conserved 3-amino-6-hydroxy-2-piperidone (Ahp). Variable amino acids are indicated by R. The side chain R_1_ in cyanopeptolins may consist of one, two or three units.

Anabaenopeptins (APs) and cyanopeptolins (CYPs) belong to cyclic nonribosomal oligopeptides (NRPs) produced by cyanobacteria. Characteristic of the APs is a ring formed by five amino acid residues, including a conserved Lys. The ring part of the molecule is connected to the one amino acid residue side chain through an ureido bond [2] (Figure 1A). Except for the conserved Lys, the amino acid residues are variable [3]. CYPs, which belong to the depsipeptides, have a six amino acid residue ring structure, a conserved 3-amino-6-hydroxy-2-piperidone (Ahp) amino acid residue and a side chain with variable length [4] (Figure 1B). In CYPs, the Ahp is conserved. In addition, they may have several variable non-proteogenic organic acids in their structure. Consequently, the structure diversity among the cyanobacteria CYPs is astonishing and several structural analogs of the compounds are known under different names [3]. APs or CYPs have been found in several marine, brackish and freshwater cyanobacteria genera, *i.e.*, *Dolichospermum*, *Lyngbya*, *Microcystis*, *Nodularia*, *Nostoc*, *Oscillatoria* and *Planktothrix* (references in [5,6,7]).

Reported bioactive properties of APs and CYPs are often related to inhibition of serine proteases and protein-serine/threonine phosphatases [5,7]. These enzymes are responsible for the regulation of several vital physiological metabolic processes [8,9]. Additionally, inhibition of protein-serine/threonine phosphatases by cyanobacteria NRPs was associated with tumor promotion [10,11].

The biological function of NRPs is unclear, but a reaction to top-down regulation by parasites of cyanobacteria remains a convincing hypothesis to be tested [12]. Similarly, the ecological roles of APs and CYPs are unresolved. Bioaccumulation of APs was shown in aquatic organisms [13,14] and negative effects of CYPs on proteases in grazers were documented [15,16]. Regardless of their biological and ecological functions, the remarkable NRP diversity in cyanobacteria and the uniqueness of NRP profiles in individual strains render them highly applicable as biomarkers, even at subspecies levels [12].

The morphologically similar and closely related cyanobacteria genera *Snowella* and *Woronichinia* belong to the family Coelosphaeriaceae in the order Synechococcales [17]. Phylogenetic studies of species in this family are rare and the relationships between species in the family remain uncertain [18,19]. However, a division of morphologically separable *Snowella* and *Woronichinia* species was verified by molecular methods [20]. Both genera occur worldwide in freshwater and brackish environments [17,21]. *Woronichinia* sometimes causes blooms [22,23,24,25], even associated with fish kills [26]. Due to scarcity of strains, most of the reports on secondary metabolites and potential toxicity concern field samples [23,25,27,28,29]. Genes coding for NRPs were confirmed in *Woronichinia* *naegeliana* and NRPs-like gene sequences were reported from *Snowella* *litoralis* [30].

The aim of this study was to increase the limited knowledge of cyanobacteria in Coelosphaeriaceae by a polyphasic characterization of a species in the family. The morphology, phylogeny and NRP profile of the cyanobacterium were studied. Furthermore, the bioactive potential of its NRPs was explored through inhibition of serine proteases and protein-serine/threonine phosphatases. The studies were done with an established single species culture.

## 2. Results

### 2.1. Morphologic and Phylogenetic Characterization

**Figure 2:**
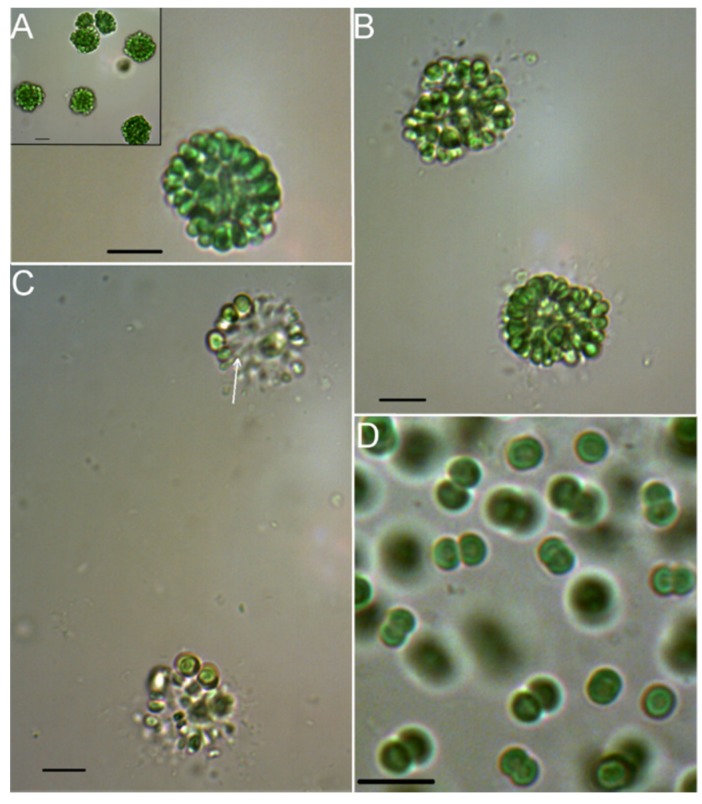
Microphotographs of the isolated coelosphaeran cyanobacterium (strain 06S067) (**A**) in a culture maintained for three months (insert five months); (**B**) outer mucilage layer (culture maintained for five months); (**C**) gelatinous stalks (arrow) visible in decomposing colonies; and (**D**) in a culture maintained for 2.5 years. The scale bars are 10 µm.

The newly isolated strain 06S067 grew in spherical and irregularly shaped colonies, also forming subcolonies (Figure 2A), which were rigid and 15–22 µm in diameter. Cells were 3 µm wide, 3–5 µm long, dark, olive or clear green, obtuse and ovoid with somewhat flattened sides, radially and tightly arranged, especially in the outer layer. Gas vacuoles were not observed. An outer mucilage layer was not clearly visible in the light microscope (Figure 2B). Gelatinous stalks were only observed in decomposing colonies and appeared unbranched (Figure 2C). Based on the described morphological features, the species resembled *Woronichinia compacta* [21,31]. After one year in culture, only cell pairs were formed (Figure 2D). When growing in pairs, the cells were 2–4 µm wide and 2–3 µm long. Solitary cells were never observed. Biomass for the phylogenetic analyses was obtained from a culture maintained for ~4 years. Analyses of NRP structures and activities were done on biomass from a culture maintained for ~3 years.

**Figure 3:**
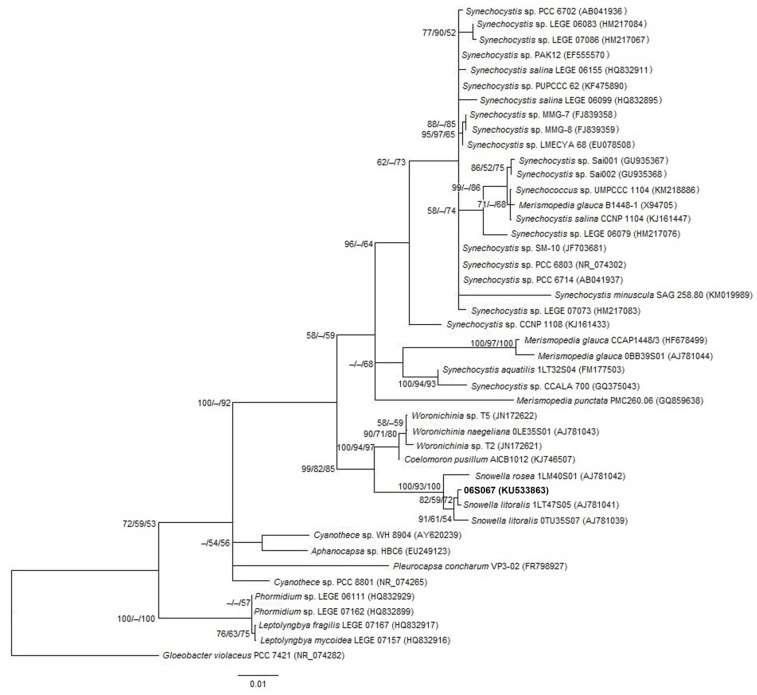
Maximum likelihood tree based on partial 16S rRNA sequences. The analyzed strain 06S067 (895 bp) in bold. Other 16S rRNA sequences were retrieved from GenBank, accession numbers in brackets. Bootstrap values >50% are shown at the nodes for neighbor-joining/maximum parsimony/maximum likelihood analyses. The scale bar indicates number of nucleotide substitutions per site. *Gloeobacter violaceus* was used as an out-group.

The length of the sequenced partial 16S rRNA gene of strain 06S067 was 895 bp. It shared 98.7%–99.8% partial 16S rRNA gene sequence similarity with *Snowella* species, 96.8%–96.9% with *Woronichinia* species and 96.9% with *Coelomoron pusillum* (Figure 3). The *Snowella* and *Woronichinia* species, including strain 06S067, shared 96.1%–96.9% 16S rRNA gene sequence similarity. Tree topologies obtained from neighbor-joining, maximum parsimony and maximum likelihood analyses were compatible.

### 2.2. Analyses of Nonribosomal Oligopeptides

Mass spectra (MS) and MS/MS fragmentation spectra indicative of seven APs were identified in the reversed phase fractions of strain 06S067 eluted with 20%–100% methanol (Table 1). Majority of the APs were found in the 40% methanol fraction. Presence of the conserved Lys immonium ion (at mass-to-charge ratio, *m*/*z* 84) was a precondition for identification of compounds with characteristic fragmentation spectra as potential APs. Other proposed AP fragmentations at low *m*/*z* were the Phe immonium ion (*m*/*z* 120) and Arg related fragmentation ions ([Arg + 2H] at *m*/*z* 175, [CO + Arg + H] at *m*/*z* 201). The amino acid residue in exocyclic position was preliminary identified by the loss of its characteristic mass, usually as [Arg + CO] (∆ 200 Da) or [Tyr + H_2_O] (∆ 181 Da) from the pseudomolecular ion. The suggested main fragment ions by which the potential AP structures were characterized (Figures S1–S10) and LC-MS/MS chromatograms of the fractions (Figures S11–S19) are presented in the supplementary figures.

The compound at *m*/*z* 844, with an ion fragmentation spectrum indicative of AP A (anabaenopeptin A), was most abundant (identified in five of the fractions, 20%–60% methanol, Table 1) and displayed the most intense ion in the total ion chromatogram, as expressed by counted ions per units (1.6 × 10^10^ cpu; 30% methanol). Another intense signal (7.0 × 10^9^ cpu; 50% methanol) was represented by the compound at *m*/*z* 837, with an ion fragmentation spectrum indicative of AP B (anabaenopeptin B). This *m*/*z* was identified in three of the fractions (40%–60% methanol, Table 1). The compound at *m*/*z* 851 with an ion fragmentation similar to AP F (anabaenopeptin F) was identified in one fraction (60% methanol, Table 1). An ion fragmentation spectrum indicative of oscillamide Y (OSC Y) at *m*/*z* 858, was found in three fractions (30%–50% methanol, Table 1). Three ion fragmentation spectra indicated AP structures, whereas their *m*/*z* (803, 810, and 828) have not previously been described for APs (*cf.* [5]). Ion fragmentation spectra indicative of CYP-like compounds were also identified in the 06S067 fractions. An ion fragmentation spectrum of a suggested CYP-like compound is illustrated in the supplementary Figure S10.

### 2.3. Analyses of Enzyme Inhibitory Activity

The 30% and 40% methanol fractions showed inhibitory activity against carboxypeptidase A and a consistent decreasing activity with increasing fraction polarity (Table 2). In these fractions, three [M + H]^+^ ions (*m*/*z* 810, 844, and 858) and their fragmentation spectra indicative of APs were identified (Table 1). The strongest activity against carboxypeptidase A (in the 30% methanol fraction, Table 2) coincided with the most intense ion signal of the compound at *m*/*z* 844. Inhibitory activity against trypsin was potent in the 60% methanol fraction (Table 2), in which [M + H]^+^ ions (*m*/*z* 752, 803, 837, 844, and 851) and their fragmentation spectra suggested the presence of five different APs (Table 1). In this fraction, the compounds at *m*/*z* 803 and 837 had the most intense ions. Activity against trypsin also overlapped with fractions in which fragmentation spectra of tentative CYP-like compounds were identified. Weak activity against protein phosphatase 1 was observed in the 40%–50% methanol fractions (Table 2). In these fractions, four [M + H]^+^ (*m*/*z* 803, 837, 844, and 858) and their fragmentation spectra indicative of APs were identified (Table 1). The 30%–50% methanol fractions showed weak activity against protein phosphatase 2A (Table 2). These three fractions all contained the compounds at *m*/*z* 844 and 858. Inhibitory activity against chymotrypsin and thrombin was potent in some of the fractions but the activity increased with fraction polarity or was non-significant. The activity against elastase was non-significant (Table 2).

## 3. Discussion

### 3.1. Morphology and Phylogeny of Strain 06S067 

The newly isolated strain 06S067 showed morphologic features typical of *Woronichinia*, *i.e.*, colonies, as well as subcolonies, with tightly, radially arranged, obtuse, ovoid, and large cells. Main morphologic characteristics separating 06S067 from the genus *Snowella* were its compound colonies, with tightly packed cells and unbranched stalks, which were not clearly visible in the light microscope. Morphologically, strain 06S067 was identified as *Woronichinia compacta* (*W. compacta*) [21,31]. A recent increase of this species in coastal areas of the Baltic Sea was associated with eutrophication [32]. The phenotypic variability of *W. compacta* is poorly known [31] and its taxonomy unclear [18]. Considering the firm colonies, their homogeneous centers and dark green color, strain 06S067 also matched the description of *Woronichinia obtusa* (*W. obtusa*) [18]. Cyanobacteria from the genus *Woronichinia* reproduce by releasing solitary cells [21], but single cells were never observed in 06S067. However, in *W. obtusa* colonies were characterized as firm, not easily releasing cells, even under a cover glass [18]. Similarly to described *Snowella* and *Woronichinia* strains [20], 06S067 lost its colony form in culture. In the disintegrated form, cells were attached in pairs and resembled a described non-colony form of *Snowella* [20], although with larger cells.

In the phylogenetic analyses, *Snowella* and *Woronichinia* species, including strain 06S067, clustered with high partial 16S rRNA gene sequence similarities. In previous studies, *Snowella* and *Woronichinia* shared <95% 16S rRNA gene sequence similarity [20,33], whereas the similarity between *Snowella* and *Woronichinia* in this study was >95%. Thus, inclusion of strain 06S067 appeared to increase the phylogenetic similarity of *Snowella* and *Woronichinia*. Considering 16S rRNA gene sequences, 95% similarity was considered an upper boundary for genus definition [17]. Strain 06S067 shared highest partial 16S rRNA gene similarity with *Snowella litoralis* (*S. litoralis*) strain 1LT47S05. However, the strain lacked features such as visible stalks, spherical, distant and pale green cells, which are typical of *S. litoralis* [18,21]. Available sequences of *Snowella* and *Woronichinia* species remain scarce (nine 16S rRNA sequences of cultured species in GenBank, 16 March 2016), which can contribute to misleading identification [34]. Among the coelosphaeran species phylogenetically characterized so far, strain 06S067 is the only one isolated from a saline habitat, which also might have influenced the phylogenetic results. However, a complete revision of the family was recently recommended [19].

### 3.2. Nonribosomal Oligopeptides Produced by Strain 06S067

In this study, the production of APs was indicated in an established single species culture of a coelosphaeran cyanobacterium. Potentially, as indicated by the characteristic losses from the pseudomolecular ions of suggested APs, Arg or Tyr occurred in the exocyclic position. Preliminary identification of fragments in ion spectra indicated that several of the suggested APs differed in only one amino acid residue, implying that they were structurally conserved. Some of the ion fragmentation spectra appeared to correspond to certain parts of APs. The *m*/*z* values and associated fragmentation spectra of three pseudomolecular ions suggested APs that have not previously been reported (*cf.* [5]). Fractions of strain 06S067 also contained ion fragmentation spectra indicative of CYP-like compounds. Of the species in Coelosphaeriaceae, analyses of NRPs have so far mainly been done in environmental samples of *Woronichinia* [23,25,27,29]. Therefore, a direct comparison of the proposed 06S067 NRP profile with other coelosphaeran species is still of limited usefulness. In bloom samples dominated by *W. naegeliana* one AP, OSC B, as well as CYPs, microginins, and microcystin-LR were identified [29]. Microcystins were reported in *Snowella lacustris* [28], but other NRPs have rarely been associated with this genus. Potential NRPs genes were reported in *S. litoralis* strain 1LT47S05 [30], which according to the phylogenetic analyses was most similar to strain 06S067. Among *Snowella* and *Woronichinia*, CYPs [29] or CYP gene clusters [30] have only been identified in *Woronichinia*.

Analyses of ion fragmentation spectra, which indicated the sequence of the amino acid residues, combined with structural information of known APs and CYPs provided a preliminary structure characterization. However, confirmation of the amino acid residues, as well as the stereochemistry of the molecules, requires additional techniques. Nonetheless, the preliminary structures indicated a production of diverse NRPs by the strain. As the NRP profile is an important chemotaxonomic marker, it will certainly be of use in future studies of intraspecies differences within Coelosphaeriaceae phenotypes.

### 3.3. Enzyme Inhibitory Activity of Fractionated Extracts from Strain 06S067

As the composition of the 06S067 fractions was complex, it was not possible to sort out one responsible compound for the observed enzyme inhibitory activities. In addition, natural protease inhibitors normally act non-specifically and thus often inhibit several related enzymes [35]. Both APs [5,13,36,37,38] and CYPs (e.g., activities reported in [7]) have a striking ability to inhibit various types of enzymes. APs are the only known cyanobacteria peptide inhibitors of the pancreatic metalloexopeptidase carboxypeptidase A [5,37,39,40]. Among the proposed APs in strain 06S067, only AP B was previously reported to inhibit trypsin [41]. However, not all 06S067 fractions containing the compound at *m*/*z* 837, indicative of AP B, showed activity against trypsin. Furthermore, the 50% methanol fraction, in which *m*/*z* 837 displayed the most intense ion signal, did not show any trypsin inhibitory activity. Trypsin inhibition was reported in CYPs [40,42] and as the fractions with activity against trypsin also contained intense signals of potential CYP-like compounds, these NRPs were an alternative cause for the observed activity.

Weak activity against protein phosphatase 1 was observed in 06S067 fractions containing compounds with pseudomolecular ions at *m*/*z* 803, 837, 844 and 858, and fragmentation spectra indicative of AP 802, A, B and OSC Y, of which all the previously identified APs (AP A, B, OSC Y) are known protein phosphatase 1 inhibitors [5,13,41,43]. The activity against protein phosphatase 2A of fractions containing compounds with pseudomolecular ions at *m*/*z* at 844 and 858 was weak. Some fractions of 06S067, in which [M + H]^+^ *m*/*z* and their fragmentation spectra indicated the presence of both APs and CYP-like compounds, showed potent but non-significant or inconsistent activity against thrombin. Inhibition of thrombin seems to be rare among the APs, but was shown in some CYPs [7,44]. None of the fractions of strain 06S067 showed significant activity against elastase. However, several CYPs were described as elastase inhibitors [7,44,45] and activity against this enzyme was also reported for two APs [44]. Although fractions containing several compounds were used in the inhibition assays, the significant activity against carboxypeptidase A and trypsin implied a strong bioactivity of at least some of the compounds present.

The seemingly ubiquitous presence of NRPs in cyanobacteria is intriguing, here indicated by a rich diversity also in a less studied cyanobacteria group. The biological and ecological roles of these compounds are yet to be discovered. Potentially, cyanobacteria NRPs are elements of a defense mechanism against parasitic rhizoids, which penetrate cyanobacteria cells and secrete proteases [12]. Grazers experience negative side effects of the NRP enzyme inhibitors, as these inhibit proteolytic enzymes in the digestive tracts of the organisms [14,15,16], or enzymes involved in molting [46]. The APs have so far been proven to accumulate in mussels and crustaceans (whole tissue) [14]. They were also detected in four fish species (muscle and visceral tissue), a frog (visceral tissue) and a snail (whole tissue) [13]. The consequences of this AP bioaccumulation remain to be solved, but indicate that at high cyanobacteria densities, the NRPs may have effects at the ecosystem level. This can be of great significance considering the global expansion of cyanobacteria [47].

## 4. Conclusions

Here, a polyphasic identification of a coelosphaeran cyanobacterium in an established culture from a saline habitat was performed for the first time. According to the morphology, the strain was identified as *Woronichinia compacta*. The phylogenetic analyses placed the strain in close relationship with *Snowella* species. Analyses by LC-MS/MS provided a strong indication of AP, and potentially also CYP production by the strain. Preliminary identification of the NRP structures indicated a rich chemical diversity. Enzyme inhibition activities of the fractionated extracts supported an AP and CYP production. This bioactivity has probable ecological relevance in aquatic habitats considering the global expansion of cyanobacteria. Studies of species in the family Coelosphaeriaceae are important with regard to the cosmopolitan distribution, potential toxicity and required phylogenetic revision of the family. Despite the polyphasic characterization, considering morphology, phylogeny and chemical properties of the coelosphaeran species presented here, the limited knowledge of Coelosphaeriaceae impeded a definite identification. Thus, the full potential of the results will be achieved when knowledge concerning the same, or similar, species increases.

## 5. Materials and Methods

### 5.1. Cultivation

The studied species was single colony isolated from a sample collected from a brackish (5 psu) rock pool in the NW Åland outer archipelago (60°19.933 N, 19°33.476 E) in July 2011. The morphology of the species was studied using an inverted light microscope (Leica DM IRB, Leica Microsystems, Wetzlar, Germany) and it was identified based on criteria described in [18,21,31]. The strain was non-axenically cultivated as a monospecies culture in sterile liquid Z8 medium with added nitrogen ([48] based on [49]) in artificial seawater (6 psu), at a 16:8 h light:dark cycle (50 µEm^2^·s^−1^) at 21 °C. The culture was maintained by transferring an inoculum to sterile, fresh liquid medium (ratio 1:4) every fourth week. The strain, 06S067, was deposited at the Culture Collection of Northern Poland, Institute of Oceanography, University of Gdańsk. Material for enzyme inhibition assays and analyses of NRPs was harvested from a 5 L culture by filtration (Whatman GF/A, GE Healthcare UK, Amersham, Bucks, United Kingdom), freeze-dried (Christ Alpha 1–4 LSC, Osterode, Germany) and stored at −18 °C.

### 5.2. DNA-Extraction and PCR

DNA was extracted with FastDNA SPIN Kit for Soil (MP Biomedicals, Warszawa, Poland) according to manufacturer’s instructions. Presence and condition of the extracted DNA was confirmed with 1% agarose (Agaroza LE Standard, DNA Gdańsk, Gdańsk, Poland) gel electrophoresis. The region containing 16S rRNA + ITS + 5′ end of the 23S rRNA gene in the extracted DNA was amplified using the primers CYA359F [50] and 23S30R [51]. The amplification was done in a reaction mixture containing 0.1 µL (100 µM) of each primer, 12.5 µL MyTaq Red Mix polymerase (Bioline, London, United Kingdom) and 2 µL (~200 ng) DNA sample. Final volume of the reaction mixture was 25 µL. The PCR conditions were as in [52], but annealing temperature was changed to 57 °C. Amplification was done in a Mastercycler (Eppendorf, Hamburg, Germany) and the PCR product was purified with a Clean-Up Kit (A & A Biotechnology, Gdynia, Poland). Presence and the condition of the PCR product were controlled with 1% agarose gel electrophoresis. The PCR product was sequenced (Genomed, Warzsawa, Poland) using the CYA359F primer [50] to obtain the partial 16S rRNA gene sequence. The partial 16S rRNA sequence of strain 06S067 was deposited to GenBank (accession number KU533863).

### 5.3. Phylogenetic Analyses

The 06S067 partial 16S rRNA gene sequence was compared to similar (>93% identity) 16S rRNA sequences based on a standard nucleotide BLAST search (basic local alignment search tool) [53], excluding sequences from uncultured/environmental samples. The 16S rRNA sequences were aligned with ClustalX (Version 2.1, Conway Institute UCD, Dublin, Ireland, 2010) [54]. Pairwise alignments were done using the slow-accurate option, gap opening and gap extension penalties were set to 10 and 0.10 respectively. Matches and mismatches among the bases were weighted using the IUB weight matrix. Multiple alignments were done with the gap opening penalty 10 and gap extension penalty 0.20. Merging of the sequences was delayed from the multiple alignments if their differences were >25%. Based on the nucleotide BLAST search, 43 16S rRNA sequences shared >93% identity with the 06S067 partial 16S rRNA sequence. One (*Gloeothece* sp. PCC 6909, strain CCAP 1480/4, accession number HE975009) of these 43 sequences was truncated and therefore eliminated from further analyses. Nonhomologous regions of the 42 aligned 16S rRNA sequences were manually deleted in BioEdit (Version 7.2.5, Ibis Biosciences, Carlsbad, CA, USA, 2013) [55]. The phylogeny of the edited, aligned 16S rRNA sequences was studied in the program PHYLIP (Version 3.695, University of Washington, Seattle, WA, USA, 2005) [56]. The 16S rRNA sequences were bootstrapped using the Seqboot protocol and used for constructing trees based on neighbor-joining [57], maximum parsimony and maximum likelihood analyses. For the neighbor-joining tree, 1000 bootstrap replicates were used, distance matrices (with Jukes and Cantor distance [58]) were calculated using the Dnadist protocol and the neighbor-joining trees constructed using the Neighbor protocol. Maximum parsimony trees were constructed with 1000 bootstrap replicates using the Dnapars protocol. The trees based on maximum likelihood were constructed with 100 bootstrap replicates using the Dnaml protocol, with the branch lengths iterated and global rearrangements done. Final trees were chosen based on the majority rule in the Consensus protocol. The input order of species was randomized in the analyses and *Gloeobacter violaceus* (strain PCC 7421, accession number NR_074282) was used as an out-group. The final tree was edited in FigTree (Version 1.4.2, University of Edinburgh, Edinburgh, United Kingdom, 2014) [59].

### 5.4. Biomass Extraction

The freeze-dried biomass (including filter) was crude extracted in a glass vial in 80% ethanol (*v*/*v*, 50 mL, Altia, Rajamäki, Finland) for 1 h on a platform mixer (14 rpm, Thermolyne VARI-MIX, Barnstead, Dubuque, IA, USA). The extract was centrifuged (3220 *g*, 10 min, 20 °C, Allegra X-12R, Beckman-Coulter, Fullerton, CA, USA) and the supernatant collected, after which the crude extraction was repeated. The supernatants were vacuum filtered (GHP Acrodisc GF 25 mm syringe filter with GF prefilter and 0.45 µm GHP membrane, Pall Life Sciences, Port Washington, NY, USA). Ethanol was evaporated from the filtrate under pressure in a rotavapor (RE100, Bibby, Stone, Staffordshire, United Kingdom) at 50 °C, remaining water was freeze-dried. The dry crude extract was stored at −18 °C for one month, after which it was further extracted by solid phase extraction (SPE) with C18 SPE cartridges (1 g C18(EC) 3 mL, ISOLUTE, Mid Glamorgan, United Kingdom). The crude extract was dissolved in 2 mL 0.5% dimethyl sulfoxide, DMSO (Riedel-De-Haën, Seelze, Germany) in ultrapure water (purified to 18.2 MΩcm on a Milli-Q Synthesis system, Millipore, Molsheim, France) and remaining residues were removed by filtering (GHP Acrodisc GF 25 mm syringe filter with GF prefilter and 0.45 µm GHP membrane, Pall Life Sciences, Port Washington, NY, USA). The SPE-cartridge was conditioned with 100% methanol (HPLC Grade, Rathburn, Walkerburn, Scotland) and equilibrated with ultrapure water. The sample (2 mL) was loaded and passed through the cartridge at a flow rate of 50 µL·s^−1^. The fraction unbounded to the resin was washed with ultrapure water and the cartridge was air dried for 15 min. The retained analytes were eluted with aqueous methanol (HPLC Grade, Rathburn, Walkerburn, Scotland) solutions with increasing concentration of organic solvent (10, 20, 30, 40, 50, 60, 70, 80, 90, and 100%) at the flow rate 25 µL·s^−1^. Acquired fractions were dried under N_2_-flow, redissolved in 0.5% DMSO and divided into two portions for analyses of NRPs and for enzyme inhibition assays.

### 5.5. Analyses of Nonribosomal Oligopeptides

The collected fractions were redissolved in 30% aqueous methanol (HPLC Grade, Merck, Darmstadt, Germany), centrifuged (10,000 *g*, 15 min, 22 °C, Centrifuge 5418R, Eppendorf, Hamburg, Germany) and supernatants transferred to chromatographic vials. The fractions were analyzed with liquid chromatography tandem mass spectrometry (LC-MS/MS) on Agilent 1200 (Agilent Technologies, Waldboronn, Germany) coupled online to a hybrid triple quadrupole/linear ion trap mass spectrometer (QTRAP5500, Applied Biosystems, Sciex, Concord, ON, Canada) as in [60]. NRP structures were studied with the QTRAP LC-MS/MS system equipped with a turbo ion source (550 °C, 5.5 kV). Analyses were run in positive mode by the information-dependent acquisition method (IDA). Enhanced product ion spectra (EPI) were acquired at the interval 50–1000 Da with 50 V collision energy and 20 V collision energy spread. Declustering potential was 80. Data were gathered and processed with Analyst QS (Version 1.5.1, Applied Biosystems/MDS Analytical Technologies, Concord, ON, Canada, 2008), PeakView (Versions 2.1 and 2.2, AB Sciex, Concord, ON, Canada, 2014). The collected ion fragmentation spectra of peptides produced by the strain were compared to known AP ion fragmentation spectra (*cf.* [5]). In potential ion fragmentation spectra, fragments were identified by comparing their *m*/*z* to those of the possible fragment ions of the assumed APs.

### 5.6. Enzyme Inhibition Assays

The SPE-fractions were diluted (×1, ×10, ×100, ×1000, and ×10,000) in 0.5% DMSO. Enzyme inhibition assays were performed as colorimetric assays in 96-well plates. The diluted fractions, enzyme, substrate and reaction buffer were added to each well. Dilutions (in duplicate) of standard inhibitors were used as positive controls and for calculation of calibration curves. Each assay included two negative controls and four zero inhibitor concentrations. After incubation, the absorbance of the solutions was measured (Opsys MR microplate reader, Dynex Technologies, Chantily, VA, USA, or Varioskan Flash, Thermo Scientific, Vantaa, Finland).

Inhibition of standard inhibitors and fraction dilutions was calculated as

A_0_ − A_I_/A_0_, (1)

where A_0_ is the mean absorbance of zero inhibitor, and A_I_ is the mean absorbance of standard inhibitor or fraction. Fractions with an inhibitory activity that differed from mean inhibition with a 95% confidence interval were considered significant. Dose–response curves for standard inhibitors and fractions with significant inhibition were constructed with linear, loglinear or quadratic regression models using the program MyCurveFit (MyAssays Ltd., Sussex, United Kingdom, 2015) [61]. Accuracy of the applied regression model was evaluated by mean percent relative error between measured and back-calculated inhibition. The IC_50_ values were determined form the dose–response curves.

#### 5.6.1. Carboxypeptidase A

The carboxypeptidase A inhibition assays were modified from described methods [62,63]. Carboxypeptidase A from bovine pancreas (Sigma-Aldrich, St. Louis, MO, USA) was dissolved (50 µg·mL^−1^) in buffer solution (50 mM Tris-HCl, Sigma-Aldrich, St. Louis, MO, USA, pH 7.5). Six concentrations (1, 2, 5, 10, 25, 50, 75, and 100 µg·mL^−1^) of carboxypeptidase inhibitor from potato tuber (Sigma-Aldrich, St. Louis, MO, USA) were used. The substrate *N*-(4-methoxyphenylazoformyl)-Phe-OH (Bachem, Bubendorf, Switzerland) was dissolved (0.5 mM) in 1% DMSO. Inhibitor (10 µL) or fraction dilution (10 µL), enzyme (10 µL) and buffer (160 µL) were added to the wells and the plate was preincubated (Binder incubator BD 53, Tuttlingen, Germany) 5 min at 37 °C. Substrate (20 µL) was added to the wells and the plate was incubated at 37 °C. The absorbance at 350 nm was read after 5 min.

#### 5.6.2. Chymotrypsin

Chymotrypsin inhibition assays were performed as described [38], with following modifications: the pH of the buffer solution (0.5 M Tris-HCl, 1 M NaCl, Sigma-Aldrich, St. Louis, MO, USA, and 0.01 M CaCl_2_, Riedel-De-Haën, Seelze, Germany) was set to 7.5 and no additional HCl was added, also the absorbance (405 nm) was measured immediately after addition of the substrate *N*-succinyl-Gly-Gly-Phe-*p*-nitroanilide (Sigma-Aldrich, St. Louis, MO, USA). Chymotrypsin (Sigma-Aldrich, St. Louis, MO, USA) from bovine pancreas was used. Six concentrations (60, 100, 200, 300, 400, and 500 µg·mL^−1^) of the inhibitor aprotinin (Sigma-Aldrich, St. Louis, MO, USA) in ultrapure water were used as positive control.

#### 5.6.3. Elastase

Inhibition of elastase was based on described corresponding assays [45], with following modifications: elastase from porcine pancreas (Sigma-Aldrich, St. Louis, MO, USA) was dissolved in 0.2 M buffer solution (Tris-HCl, Sigma-Aldrich, St. Louis, MO, USA). In addition, inhibitor (10 µL) or fraction dilution (10 µL), enzyme (10 µL) and buffer solution (150 µL) were added to the wells and the plate was preincubated for 15 min at 25 °C, after which substrate (30 µL, *N*-succinyl-Ala-Ala-Ala-*p*-nitroanilide, Sigma-Aldrich, St. Louis, MO, USA) was added and the plate was incubated at 25 °C. The absorbance was read at 405 nm after 10 min. Six concentrations (5, 12.5, 25, 37.5, 50, and 125 µg·mL^−1^) of the inhibitor elastatinal (Sigma-Aldrich, St. Louis, MO, USA) in 1% DMSO were used as positive control.

#### 5.6.4. Protein Phosphatase 1

Assays for the inhibition of protein phosphatase 1 were based on described methods [64]. Protein phosphatase 1 (1.7 U·mL^−1^, New England Biolabs, Ipswich, MA, USA) was dissolved in buffer solution containing 50 mM Tris (Sigma-Aldrich, St. Louis, MO, USA, pH 7.4), 20 mg·mL^−1^ BSA (Sigma-Aldrich, St. Louis, MO, USA), 0.1 mM MnCl_2_ × 4 H_2_O (Sigma-Aldrich, St. Louis, MO, USA) and 0.2 mM DTT (Sigma-Aldrich, St. Louis, MO, USA). The substrate 4-nitrophenyl phosphate disodium salt hexahydrate (Sigma-Aldrich, St. Louis, MO, USA) was dissolved (15 mM) in buffer solution with 0.5 M Tris (pH 8.1), 20 mM MgCl_2_ × 6 H_2_O (Riedel-De-Haën, Seelze, Germany), 0.2 mM MnCl_2_ × 4 H_2_O and 1 mg·mL^−1^ BSA. Inhibitor (10 µL) or fraction dilution (10 µL), enzyme (25 µL) and substrate (200 µL) were added to the wells. The plate was incubated at 37 °C and the absorbance at 405 nm was read after 120 min. The inhibitor nodularin (Enzo Life Sciences, Lausen, Switzerland) was diluted in ultrapure water and six concentrations (0.125, 0.25, 0.5, 1, 2, and 4 ng·mL^−1^) were used as positive control.

#### 5.6.5. Protein Phosphatase 2A

Inhibition of protein phosphatase 2A was tested with MicroCystest kit (Zeu Immunotec, Zaragoza, Spain) according to manufacturer’s instructions.

#### 5.6.6. Thrombin

Thrombin inhibition assays were performed according to described methods [65], with following modifications: 0.8 mM solution in ultrapure water of the substrate *N*-*p*-tosyl-Gly-Pro-Lys-*p*-nitroanilide acetate salt (Sigma-Aldrich, St. Louis, MO, USA) was used, 160 µL buffer (0.2 M Tris-HCl, Sigma-Aldrich, St. Louis, MO, USA, pH 8.0) was added and the absorbance (405 nm) was read immediately after addition of substrate (20 µL). Thrombin (Sigma-Aldrich, St. Louis, MO, USA) from bovine plasma was used. Six concentrations (60, 120, 240, 600, 1200, and 2400 µg·mL^−1^) of the inhibitor 4-(2-aminoethyl) benzenesulfonyl fluoride hydrochloride **(**Sigma-Aldrich, St. Louis, MO, USA) in ultrapure water were used as positive control.

#### 5.6.7. Trypsin

Trypsin inhibition assays were done according to described methods [38], with following modifications: pH of the buffer solution (0.5 M Tris-HCl, 1 M NaCl, Sigma-Aldrich, St. Louis, MO, USA, and 0.01 M CaCl_2_, Riedel-De-Haën, Seelze, Germany) was set to 7.5 and to completely dissolve the substrate N_α_-benzoyl-L-arginine 4-nitroanilide hydrochloride (Sigma-Aldrich, St. Louis, MO, USA), 100% DMSO (1 mL) was added. Trypsin (Sigma-Aldrich, St. Louis, MO, USA) from porcine pancreas was used. The plate was incubated at 25 °C and the absorbance (405 nm) was read after 10 min. Six concentrations (10, 20, 30, 40, 50, and; 60 µg·mL^−1^) of the inhibitor aprotinin (Sigma-Aldrich, St. Louis, MO, USA) in ultrapure water were used as positive control.

## Figures and Tables

**Table 1:** Proposed anabaenopeptins and their mass-to-charge ratios (*m*/*z*) in methanol (MeOH) fractions of strain 06S067. The names are anabaenopeptin (AP) and oscillamide (OSC). For suggested new analogs, corresponding molecular mass is included as suffix in the name.

Fraction (% MeOH)	*m*/*z*	Proposed Anabaenopeptin
20	844	AP A
30	810	AP 809
	844	AP A
	858	OSC Y
40	803	AP 802
	810	AP 809
	828	AP 827
	837	AP B
	844	AP A
	858	OSC Y
50	752	AP fragment ^a^
	803	AP 802
	837	AP B
	844	AP A
	858	OSC Y
60	752	AP fragment ^a^
	803	AP 802
	837	AP B
	844	AP A
	851	AP F
70	637	AP fragment ^a^
80	637	AP fragment ^a^
90	637	AP fragment ^a^
100	637	AP fragment ^a^

^a^ Potential AP B related fragment.

**Table 2:** Percent inhibition of serine proteases and protein-serine/threonine phosphatases by methanol (MeOH) fractions of strain 06S067. The inhibition was calculated as fraction IC_50_ of standard inhibitor IC_50_.

Enzyme	Fraction (% MeOH)	Inhibition (%)
Carboxypeptidase A	30	13
	40	8
	50	1 ^a^
Chymotrypsin	50	5
	60	14 ^a^
Elastase	90	15 ^a^
	100	– ^b^
Protein phosphatase 1	40	0.001
	50	0.0003
Protein phosphatase 2A	30	0.003
	40	0.001
	50	0.0005
Thrombin	60	49 ^a^
	70	44
Trypsin	60	94
	70	26 ^a^

^a^ >50% mean relative error of applied regression; ^b^ Negative dose–response curve.

## Supplementary Materials

The following are available online at www.mdpi.com/2072-6651/8/4/108/s1, Figure S1. LC-MS/MS ion fragmentation spectrum of suggested anabaenopeptin A. The intensity of the ion on the *y*-axis is given as counted ions per second (cps) and the mass-to-charge ratio (*m*/*z*) on the *x*-axis. The suggested fragmented ions are at the *m*/*z* values: 844 [M + H]; 826 [M + H – H_2_O]; 816 [M + H – CO]; 798 [M + H – H_2_O – CO]; 759 [M + H – *N-*MeAla]; 741 [M + H – *N-*MeAla – H_2_O]; 681 [M + H – Tyr]; 667 [M + H – Htyr]; 663 [M + H – Tyr – H_2_O]; 649 [M + H – Htyr – H_2_O]; 637 [M + H – (CO + Tyr)]; 568 [M + H – (Htyr + Val)]; 534 [M + H – (CO + Tyr) – *N-*MeAla – H_2_O]; 405 [Lys + Val + Htyr + H]; 373 [Phe + Lys + Val – H]; 362 [*N-*MeAla + Htyr + Val + H]; 249 [Htyr + Val + H – CO]; 136 Tyr immonium ion; 120 Phe immonium ion; 114 [*N-*MeAla + CO + H]; 84 Lys immonium ion. Figure S2. LC-MS/MS ion fragmentation spectrum of suggested anabaenopeptin B. The intensity of the ion on the *y*-axis is given as counted ions per second (cps) and the mass-to-charge ratio (*m*/*z*) on the *x*-axis. The suggested fragmented ions are at the *m*/*z* values: 837 [M + H]; 819 [M + H – H_2_O]; 637 [M + H – (CO + Arg)]; 619 [M + H – (CO + Arg) – H_2_O]; 609 [M + H – (CO + Arg) – CO]; 460 [M + H – (CO + Arg) – Htyr]; 387 [Lys + Val + Htyr + H – H_2_O]; 373 [Phe + Lys + Val – H]; 231 [*N-*MeAla + Phe – H]; 201 [CO + Arg + H]; 175 [Arg + 2H]; 112 Arg ion; 84 Lys immonium ion. Figure S3. LC-MS/MS ion fragmentation spectrum of anabaenopeptin 752, a molecule tentatively related to anabaenopeptin B and fragmented ions identified by their mass-to-charge ratios (*m*/*z*). The intensity of the ion on the *y*-axis is given as counted ions per second (cps) and the mass-to-charge ratio (*m*/*z*) on the *x*-axis. The suggested fragmented ions are at the *m*/*z* values: 752 [M + H]; 734 [M + H – H_2_O]; 724 [M + H – CO]; 575 [M + H – Htyr]; 557 [M + H – Htyr – H_2_O]; 552 [M + H – (CO + Arg)]; 534 [M + H – (CO + Arg) – H_2_O]; 547 [M + H – Htyr – CO]; 524 [M + H – (CO + Arg) – CO]; 476 [Phe + Lys + (CO + Arg) + H]; 347 [Phe + Lys + Val + H – CO]; 231 [Val + Htyr + H – CO – H_2_O]; 201 [CO + Arg + H]; 175 [Arg + 2H]; 112 Arg ion; 84 Lys immonium ion. Figure S4. LC-MS/MS ion fragmentation spectrum of anabaenopeptin 637, the suggested ring part of anabaenopeptin B and fragmented ions identified by their mass-to-charge ratios (*m*/*z*). The intensity of the ion on the *y*-axis is given as counted ions per second (cps) and the mass-to-charge ratio (*m*/*z*) on the *x*-axis. The suggested fragmented ions are at the *m*/*z* values: 637 [M + H]; 619 [M + H – H_2_O]; 609 [M + H – CO]; 552 [M + H – *N-*MeAla]; 534 [M + H – *N-*MeAla – H_2_O]; 460 [*N-*MeAla + Phe + Lys + Val + H]; 442 [*N-*MeAla + Phe + Lys + Val + H – H_2_O]; 387 [Lys + Val + Htyr + H – H_2_O]; 362 [*N-*MeAla + Htyr + Val + H]; 316 [*N-*MeAla + Htyr + Val + H – CO – H_2_O]; 263 [*N-*MeAla + Htyr + H]; 231 [*N-*MeAla + Phe – H]; 120 Phe immonium ion; 114 [*N-*MeAla + CO + H]; 84 Lys immonium ion; 58 MeAla immonium ion. Figure S5. LC-MS/MS ion fragmentation spectrum of suggested anabaenopeptin F and fragmented ions identified by their mass-to-charge ratios (*m*/*z*). The intensity of the ion on the *y*-axis is given as counted ions per second (cps) and the mass-to-charge ratio (*m*/*z*) on the *x*-axis. The suggested fragmented ions are at the *m*/*z* values: 851 [M + H]; 833 [M + H – H_2_O]; 766 [M + H – *N-*MeAla]; 651 [M + H – (CO + Arg)]; 633 [M + H – (CO + Arg) – H_2_O]; 623 [M + H – (CO + Arg) – CO]; 474 [M + H – (CO + Arg) – Htyr]; 401 [Lys + Ile + Htyr + H – H_2_O]; 376 [*N-*MeAla + Htyr + Ile + H]; 361 [*N-*MeAla + Phe + Lys + H]; 201 [CO + Arg + H]; 175 [Arg + 2H]; 112 Arg ion; 84 Lys immonium ion. Figure S6. LC-MS/MS ion fragmentation spectrum of suggested oscillamide Y, and fragmented ions identified by their mass-to-charge ratios (*m*/*z*). The intensity of the ion on the *y*-axis is given as counted ions per second (cps) and the mass-to-charge ratio (*m*/*z*) on the *x*-axis. The suggested fragmented ions are at the *m*/*z* values: 858 [M + H]; 840 [M + H – H_2_O]; 830 [M + H – CO]; 755 [M + H – *N-*MeAla – H_2_O]; 695 [M + H – Tyr]; 681 [M + H – Htyr]; 677 [M + H – Tyr – H_2_O]; 651 [M + H – (CO + Tyr)]; 568 [M + H – (Htyr + Ile)]; 550 [M + H – (Htyr + Ile) – H_2_O]; 405 [M + H – Tyr – (Htyr + Ile)]; 263 [*N-*MeAla + Htyr + H]; 150 Htyr immonium ion; 120 Phe immonium ion; 114 [*N-*MeAla + CO + H]; 84 Lys immonium ion. Figure S7. LC-MS/MS ion fragmentation spectrum of the suggested anabaenopeptin 802 and fragmented ions identified by their mass-to-charge ratios (*m*/*z*). The intensity of the ion on the *y*-axis is given as counted ions per second (cps) and the mass-to-charge ratio (*m*/*z*) on the *x*-axis. The suggested fragmented ions are at the *m*/*z* values: 803 [M + H]; 785 [M + H – H_2_O]; 603 [M + H – (CO + Arg)]; 585 [M + H – (CO + Arg) – H_2_O]; 500 [M + H – (CO + Arg) – *N-*MeAla – H_2_O]; 426 [M + H – (CO + Arg) – Htyr]; 387 [Lys + Val + Htyr + H – H_2_O]; 362 [*N-*MeAla + Htyr + Val + H]; 313 [Ile/Leu + Lys + Val + H – CO]; 201 [CO + Arg + H]; 175 [Arg + 2H]; 112 Arg ion; 84 Lys immonium ion. Figure S8. LC-MS/MS ion fragmentation spectrum of the suggested anabaenopeptin 809 and fragmented ions identified by their mass-to-charge ratios (*m*/*z*). The intensity of the ion on the *y*-axis is given as counted ions per second (cps) and the mass-to-charge ratio (*m*/*z*) on the *x*-axis. The suggested fragmented ions are at the *m*/*z* values: 810 [M + H]; 792 [M + H – H_2_O]; 782 [M + H – CO]; 764 [M + H – H_2_O – CO]; 647 [M + H – Tyr]; 633 [M + H – Htyr]; 629 [M + H – Tyr – H_2_O]; 603 [M + H – (CO + Tyr)]; 585 [M + H – (CO + Tyr) – H_2_O]; 534 [M + H – Ile/Leu-Tyr]; 516 [M + H – Ile/Leu – Tyr – H_2_O]; 488 [M + H – Ile/Leu – Tyr – H_2_O – CO]; 449 [M + H – Tyr – (*N-*MeAla + Ile/Leu)]; 431 [M + H – Tyr – (*N-*MeAla + Ile/Leu) – H_2_O]; 403 [Lys + Val + Htyr – H]; 387 [Lys + Val + Htyr + H – H_2_O]; 339 [Val + Lys + Ile/Leu – H]; 263 [*N-*MeAla + Htyr + H]; 84 Lys immonium ion; 58 MeAla immonium ion. Figure S9. LC-MS/MS ion fragmentation spectrum of the suggested anabaenopeptin 827 and fragmented ions identified by their mass-to-charge ratios (*m*/*z*). The intensity of the ion on the *y*-axis is given as counted ions per second (cps) and the mass-to-charge ratio (*m*/*z*) on the *x*-axis. The suggested fragmented ions are at the *m*/*z* values: 828 [M + H]; 810 [M + H – H_2_O]; 800 [M + H – CO]; 725 [M + H – *N-*MeAla – H_2_O]; 647 [M + H – Tyr – H_2_O]; 621 [M + H – (CO + Tyr)]; 603 [M + H – (CO + Tyr) – H_2_O]; 534 [M + H – Tyr – *N-*MeAla – CO – H_2_O]; 405 [M + H – Tyr – (Hph + Val)]; 387 [Lys + Val + Hph – H]; 373 [Phe + Lys + Val – H]; 320 [M + H – Tyr – (Val + Hph + *N-*MeAla)]; 247 [*N-*MeAla + Hph + H]; 231 [*N-*MeAla + Phe – H]; 120 Phe immonium ion; 84 Lys immonium ion. Figure S10. LC-MS/MS ion fragmentation spectrum of a suggested cyanopeptolin-like compound and fragmented ions identified by their mass-to-charge ratios (*m*/*z*). The intensity of the ion on the *y*-axis is given as counted ions per second (cps) and the mass-to-charge ratio (*m*/*z*) on the *x*-axis. The suggested fragmented ions are at the *m*/*z* values: 800 [the ring structure]; 521 [Lys + Ahp + Phe + MeTyr – H_2_O – CO + H]; 420 [Ahp + Phe + MeTyr – H_2_O + H]. Figure S11. LC-MS/MS chromatogram of the 20% methanol fraction indicating the compound at *m*/*z* 844. The intensity of the ion on the *y*-axis is given as counted ions per second (cps) and the time (minutes) on the *x*-axis. Figure S12. LC-MS/MS chromatogram of the 30% methanol fraction indicating the compounds at *m*/*z* 810, 844 and 858. The intensity of the ion on the *y*-axis is given as counted ions per second (cps) and the time (minutes) on the *x*-axis. Figure S13. LC-MS/MS chromatogram of the 40% methanol fraction indicating the compounds at *m*/*z* 803, 810, 828, 837, 844 and 858. The intensity of the ion on the *y*-axis is given as counted ions per second (cps) and the time (minutes) on the *x*-axis. Figure S14. LC-MS/MS chromatogram of the 50% methanol fraction indicating the compounds at *m*/*z* 752, 803, 837, 844 and 858. The intensity of the ion on the *y*-axis is given as counted ions per second (cps) and the time (minutes) on the *x*-axis. Figure S15. LC-MS/MS chromatogram of the 60% methanol fraction indicating the compounds at *m*/*z* 752, 803, 837, 844 and 851. The intensity of the ion on the *y*-axis is given as counted ions per second (cps) and the time (minutes) on the *x*-axis. Figure S16. LC-MS/MS chromatogram of the 70% methanol fraction indicating the compound at *m*/*z* 637 and a cyanopeptolin-like (CYP-like) compound. The intensity of the ion on the *y*-axis is given as counted ions per second (cps) and the time (minutes) on the *x*-axis. Figure S17. LC-MS/MS chromatogram of the 80% methanol fraction indicating the compound at *m*/*z* 637. The intensity of the ion on the *y*-axis is given as counted ions per second (cps) and the time (minutes) on the *x*-axis. Figure S18. LC-MS/MS chromatogram of the 90% methanol fraction indicating the compound at *m*/*z* 637. The intensity of the ion on the *y*-axis is given as counted ions per second (cps) and the time (minutes) on the *x*-axis. Figure S19. LC-MS/MS chromatogram of the 100% methanol fraction indicating the compound at *m*/*z* 637. The intensity of the ion on the *y*-axis is given as counted ions per second (cps) and the time (minutes) on the *x*-axis.

## Abbreviations

The following abbreviations are used in this manuscript:

AP	anabaenopeptin
BLAST	basic local alignment search tool
CYP	cyanopeptolin
NRP	nonribosomal oligopeptide
OSC	oscillamide
PCR	polymerase chain reaction
