# Supplementary material for: Morphologic, Phylogenetic and Chemical Characterization of a Brackish Colonial Picocyanobacterium (Coelosphaeriaceae) with Bioactive Properties
Source: Toxins (Basel). 2016 Apr 12;8(4):108. doi: 10.3390/toxins8040108 (PMC4848634; doi:10.3390/toxins8040108)
Supplement: Supplementary file 1 [file toxins-08-00108-s001.pdf]

Supplementary Materials: Morphologic, Phylogenetic and Chemical Characterization of a Brackish Colonial Picocyanobacterium (Coelosphaeriaceae) with Bioactive Properties

Kerstin Häggqvist [1,*], Anna Toruńska-Sitarz [2], Agata Błaszczyk [2], Hanna Mazur-Marzec [2] and Jussi Meriluoto [1]

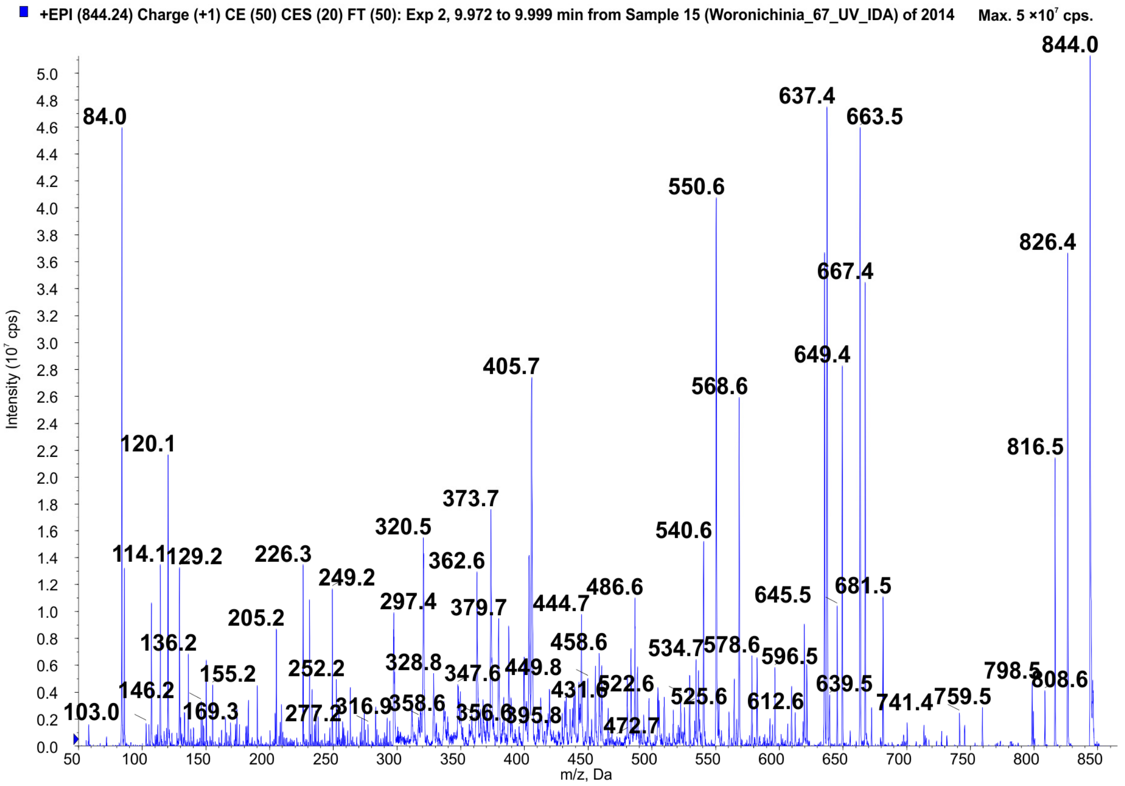

Figure S1. LC-MS/MS ion fragmentation spectrum of suggested anabaenopeptin A. The intensity of the ion on the *y*-axis is given as counted ions per second (cps) and the mass-to-charge ratio (*m*/*z*) on the *x*-axis. The suggested fragmented ions are at the *m*/*z* values: 844 [M + H]; 826 [M + H – H_2O]; 816 [M + H – CO]; 798 [M + H – H_2O – CO]; 759 [M + H – *N*-MeAla]; 741 [M + H – *N*-MeAla – H_2O]; 681 [M + H – Tyr]; 667 [M + H – Htyr]; 663 [M + H – Tyr – H_2O]; 649 [M + H – Htyr – H_2O]; 637 [M + H – (CO + Tyr)]; 568 [M + H – (Htyr + Val)]; 534 [M + H – (CO + Tyr) – *N*-MeAla – H_2O]; 405 [Lys + Val + Htyr + H]; 373 [Phe + Lys + Val – H]; 362 [*N*-MeAla + Htyr + Val + H]; 249 [Htyr + Val + H – CO]; 136 Tyr immonium ion; 120 Phe immonium ion; 114 [*N*-MeAla + CO + H]; 84 Lys immonium ion.

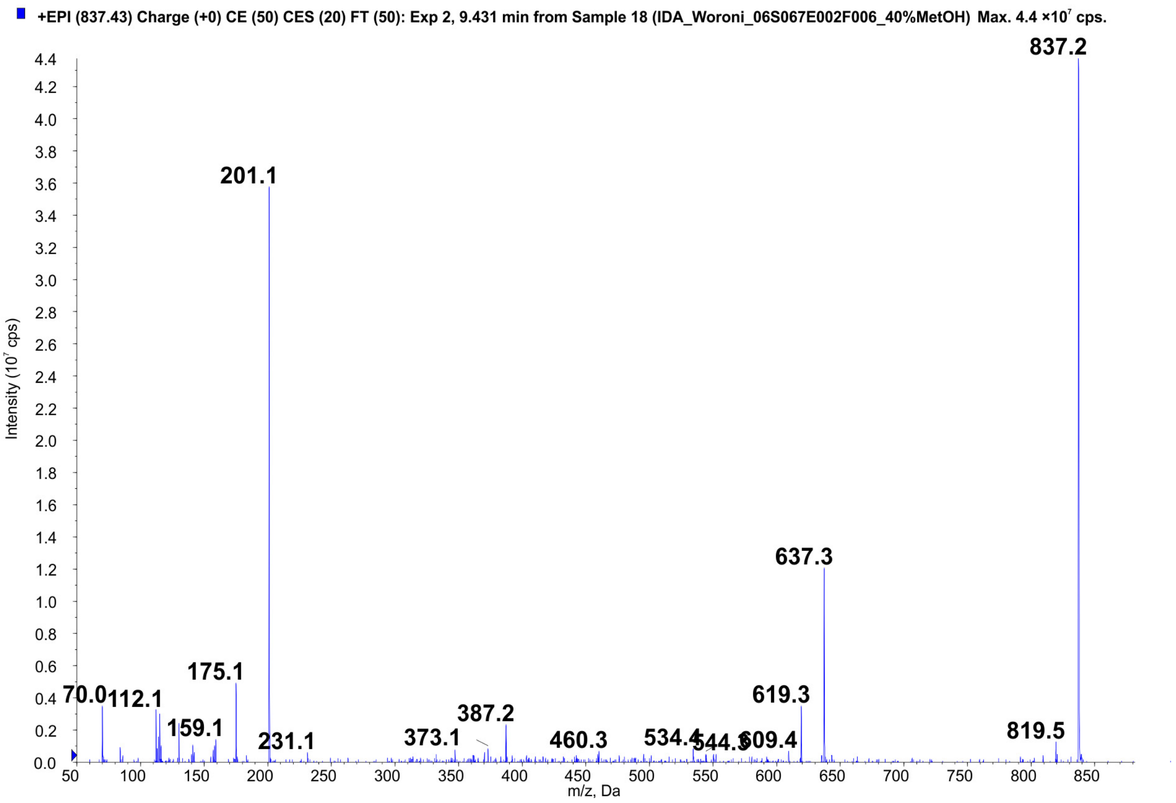

Figure S2. LC-MS/MS ion fragmentation spectrum of suggested anabaenopeptin B. The intensity of the ion on the *y*-axis is given as counted ions per second (cps) and the mass-to-charge ratio (*m*/*z*) on the *x*-axis. The suggested fragmented ions are at the *m*/*z* values: 837 [M + H]; 819 [M + H – H_2O]; 637 [M + H – (CO + Arg)]; 619 [M + H – (CO + Arg) – H_2O]; 609 [M + H – (CO + Arg) – CO]; 460 [M + H – (CO + Arg) – Htyr]; 387 [Lys + Val + Htyr + H – H_2O]; 373 [Phe + Lys + Val – H]; 231 [*N*-MeAla + Phe – H]; 201 [CO + Arg + H]; 175 [Arg + 2H]; 112 Arg ion; 84 Lys immonium ion.

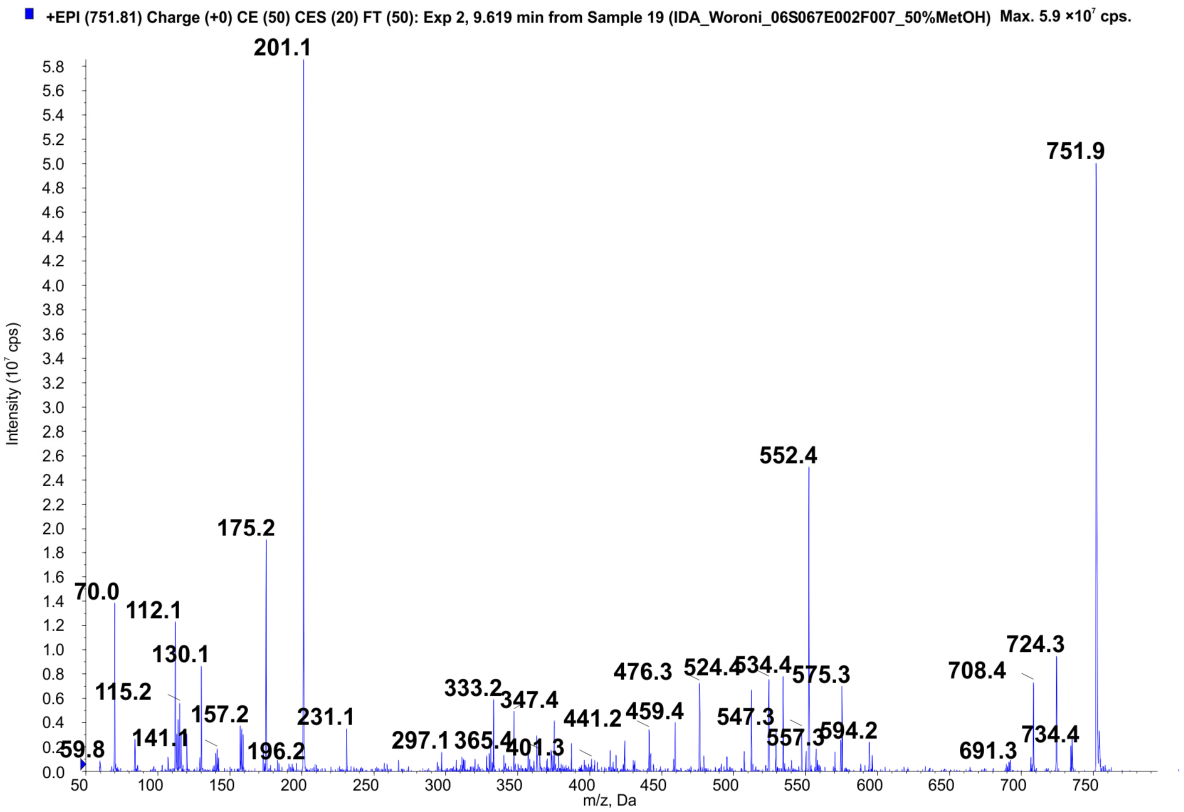

Figure S3. LC-MS/MS ion fragmentation spectrum of anabaenopeptin 752, a molecule tentatively related to anabaenopeptin B and fragmented ions identified by their mass-to-charge ratios (*m*/*z*). The intensity of the ion on the *y*-axis is given as counted ions per second (cps) and the mass-to-charge ratio (*m*/*z*) on the *x*-axis. The suggested fragmented ions are at the *m*/*z* values: 752 [M + H]; 734 [M + H – H_2O]; 724 [M + H – CO]; 575 [M + H – Htyr]; 557 [M + H – Htyr – H_2O]; 552 [M + H – (CO + Arg)]; 534 [M + H – (CO + Arg) – H_2O]; 547 [M + H – Htyr – CO]; 524 [M + H – (CO + Arg) – CO]; 476 [Phe + Lys + (CO + Arg) + H]; 347 [Phe + Lys + Val + H – CO]; 231 [Val + Htyr + H – CO – H_2O]; 201 [CO + Arg + H]; 175 [Arg + 2H]; 112 Arg ion; 84 Lys immonium ion.

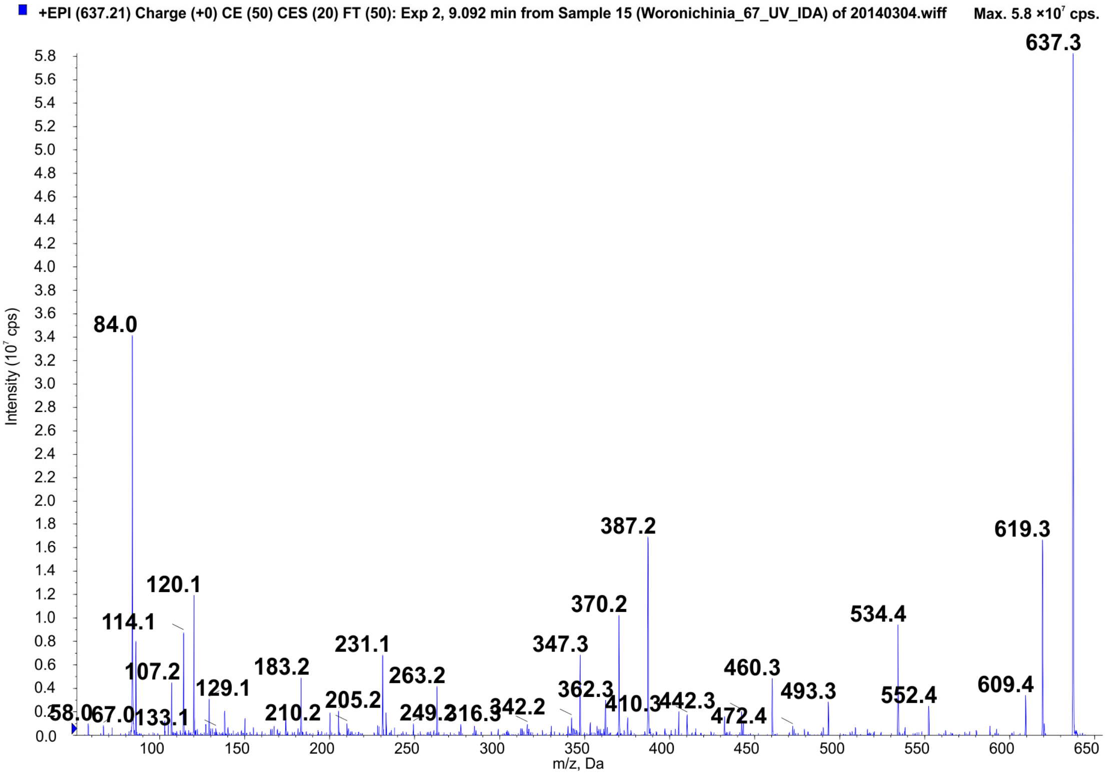

Figure S4. LC-MS/MS ion fragmentation spectrum of anabaenopeptin 637, the suggested ring part of anabaenopeptin B and fragmented ions identified by their mass-to-charge ratios (*m*/*z*). The intensity of the ion on the *y*-axis is given as counted ions per second (cps) and the mass-to-charge ratio (*m*/*z*) on the *x*-axis. The suggested fragmented ions are at the *m*/*z* values: 637 [M + H]; 619 [M + H – H_2O]; 609 [M + H – CO]; 552 [M + H – *N*-MeAla]; 534 [M + H – *N*-MeAla – H_2O]; 460 [*N*-MeAla + Phe + Lys + Val + H]; 442 [*N*-MeAla + Phe + Lys + Val + H – H_2O]; 387 [Lys + Val + Htyr + H – H_2O]; 362 [*N*-MeAla + Htyr + Val + H]; 316 [*N*-MeAla + Htyr + Val + H – CO – H_2O]; 263 [*N*-MeAla + Htyr + H]; 231 [*N*-MeAla + Phe – H]; 120 Phe immonium ion; 114 [*N*-MeAla + CO + H]; 84 Lys immonium ion; 58 MeAla immonium ion.

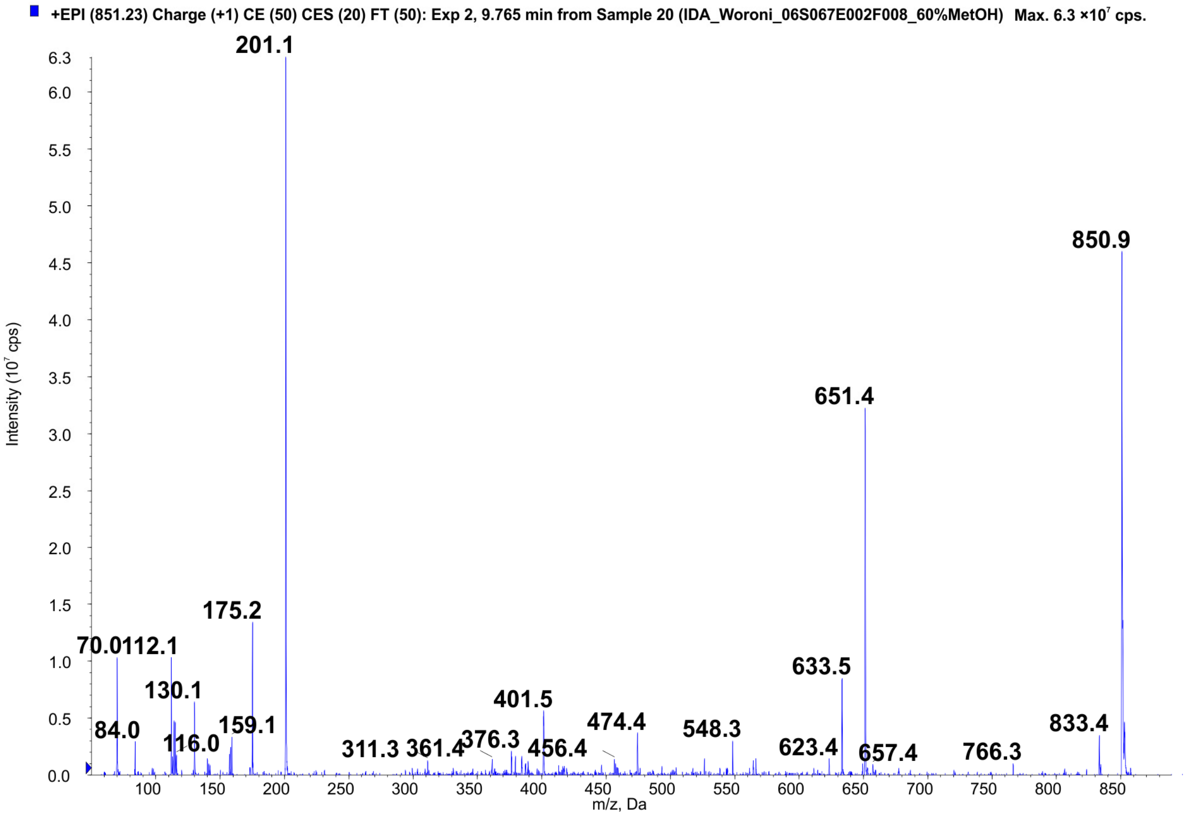

Figure S5. LC-MS/MS ion fragmentation spectrum of suggested anabaenopeptin F and fragmented ions identified by their mass-to-charge ratios (*m*/*z*). The intensity of the ion on the *y*-axis is given as counted ions per second (cps) and the mass-to-charge ratio (*m*/*z*) on the *x*-axis. The suggested fragmented ions are at the *m*/*z* values: 851 [M + H]; 833 [M + H – H_2O]; 766 [M + H – *N*-MeAla]; 651 [M + H – (CO + Arg)]; 633 [M + H – (CO + Arg) – H_2O]; 623 [M + H – (CO + Arg) – CO]; 474 [M + H – (CO + Arg) – Htyr]; 401 [Lys + Ile + Htyr + H – H_2O]; 376 [*N*-MeAla + Htyr + Ile + H]; 361 [*N*-MeAla + Phe + Lys + H]; 201 [CO + Arg + H]; 175 [Arg + 2H]; 112 Arg ion; 84 Lys immonium ion.

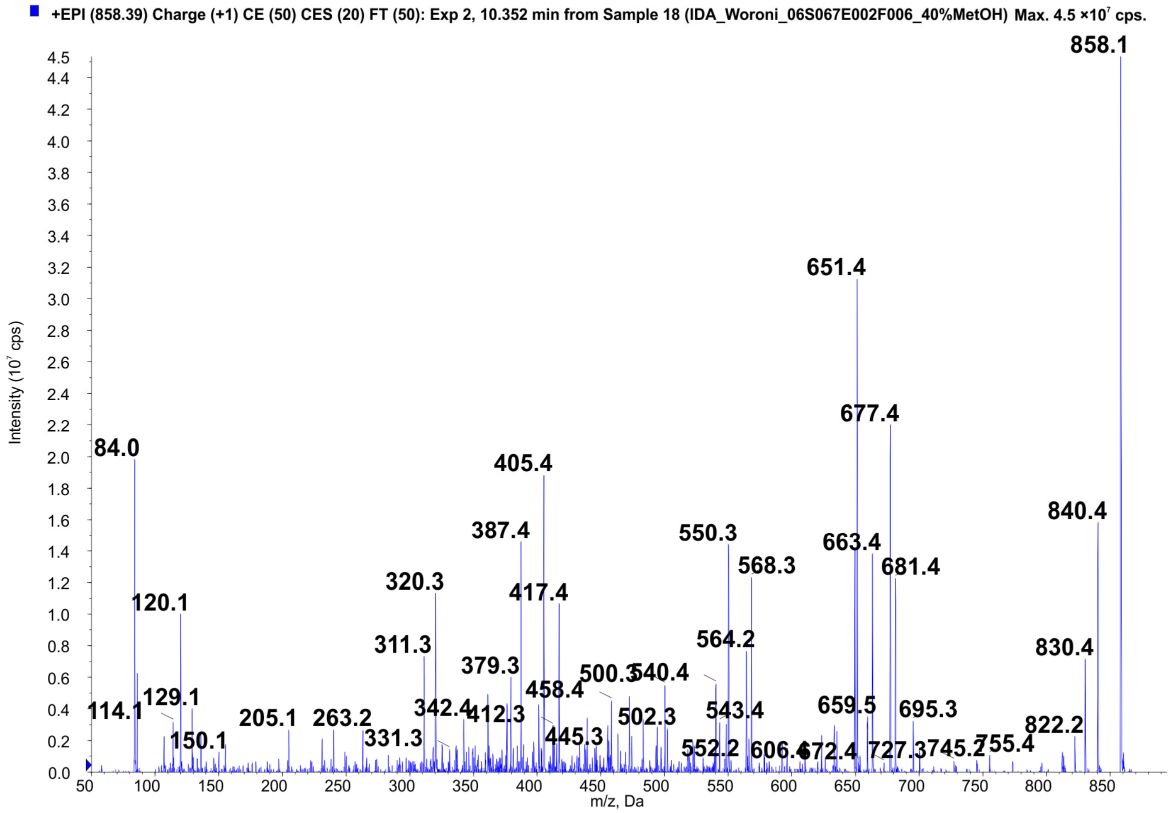

Figure S6. LC-MS/MS ion fragmentation spectrum of suggested oscillamide Y, and fragmented ions identified by their mass-to-charge ratios (*m*/*z*). The intensity of the ion on the *y*-axis is given as counted ions per second (cps) and the mass-to-charge ratio (*m*/*z*) on the *x*-axis. The suggested fragmented ions are at the *m*/*z* values: 858 [M + H]; 840 [M + H – H_2O]; 830 [M + H – CO]; 755 [M + H – *N*-MeAla – H_2O]; 695 [M + H – Tyr]; 681 [M + H – Htyr]; 677 [M + H – Tyr – H_2O]; 651 [M + H – (CO + Tyr)]; 568 [M + H – (Htyr + Ile)]; 550 [M + H – (Htyr + Ile) – H_2O]; 405 [M + H – Tyr – (Htyr + Ile)]; 263 [*N*-MeAla + Htyr + H]; 150 Htyr immonium ion; 120 Phe immonium ion; 114 [*N*-MeAla + CO + H]; 84 Lys immonium ion.

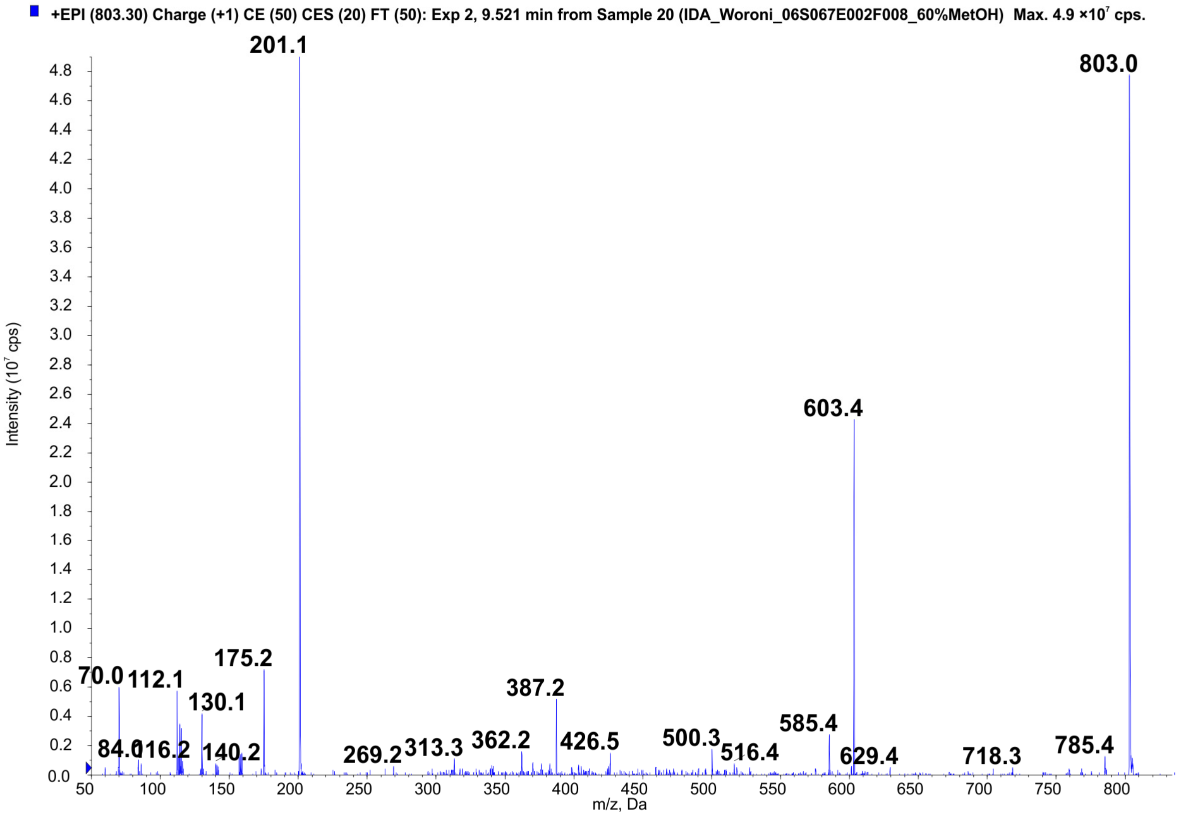

Figure S7. LC-MS/MS ion fragmentation spectrum of the suggested anabaenopeptin 802 and fragmented ions identified by their mass-to-charge ratios (*m*/*z*). The intensity of the ion on the *y*-axis is given as counted ions per second (cps) and the mass-to-charge ratio (*m*/*z*) on the *x*-axis. The suggested fragmented ions are at the *m*/*z* values: 803 [M + H]; 785 [M + H – H_2O]; 603 [M + H – (CO + Arg)]; 585 [M + H – (CO + Arg) – H_2O]; 500 [M + H – (CO + Arg) – *N*-MeAla – H_2O]; 426 [M + H – (CO + Arg) – Htyr]; 387 [Lys + Val + Htyr + H – H_2O]; 362 [*N*-MeAla + Htyr + Val + H]; 313 [Ile/Leu + Lys + Val + H – CO]; 201 [CO + Arg + H]; 175 [Arg + 2H]; 112 Arg ion; 84 Lys immonium ion.

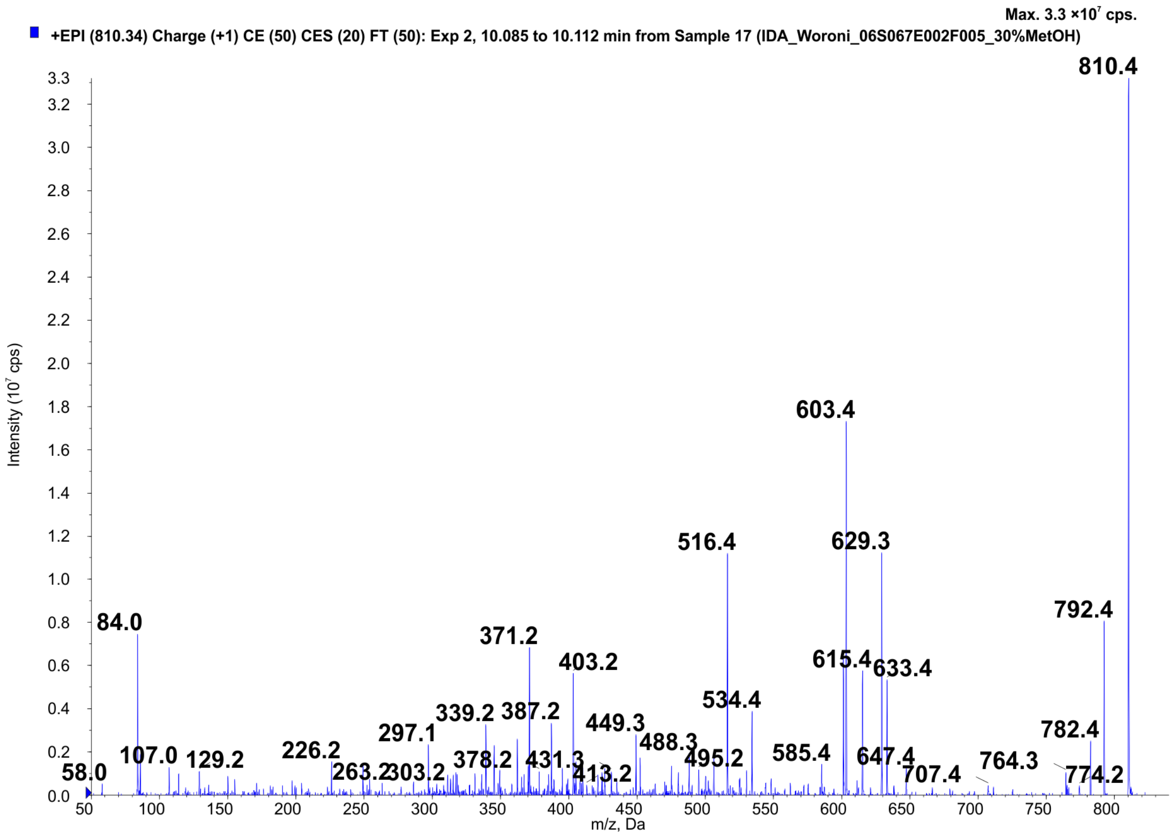

Figure S8. LC-MS/MS ion fragmentation spectrum of the suggested anabaenopeptin 809 and fragmented ions identified by their mass-to-charge ratios (*m*/*z*). The intensity of the ion on the *y*-axis is given as counted ions per second (cps) and the mass-to-charge ratio (*m*/*z*) on the *x*-axis. The suggested fragmented ions are at the *m*/*z* values: 810 [M + H]; 792 [M + H – H_2O]; 782 [M + H – CO]; 764 [M + H – H_2O – CO]; 647 [M + H – Tyr]; 633 [M + H – Htyr]; 629 [M + H – Tyr – H_2O]; 603 [M + H – (CO + Tyr)]; 585 [M + H – (CO + Tyr) – H_2O]; 534 [M + H – Ile/Leu-Tyr]; 516 [M + H – Ile/Leu – Tyr – H_2O]; 488 [M + H – Ile/Leu – Tyr – H_2O – CO]; 449 [M + H – Tyr – (*N*-MeAla + Ile/Leu)]; 431 [M + H – Tyr – (*N*-MeAla + Ile/Leu) – H_2O]; 403 [Lys + Val + Htyr – H]; 387 [Lys + Val + Htyr + H – H_2O]; 339 [Val + Lys + Ile/Leu – H]; 263 [*N*-MeAla + Htyr + H]; 84 Lys immonium ion; 58 MeAla immonium ion.

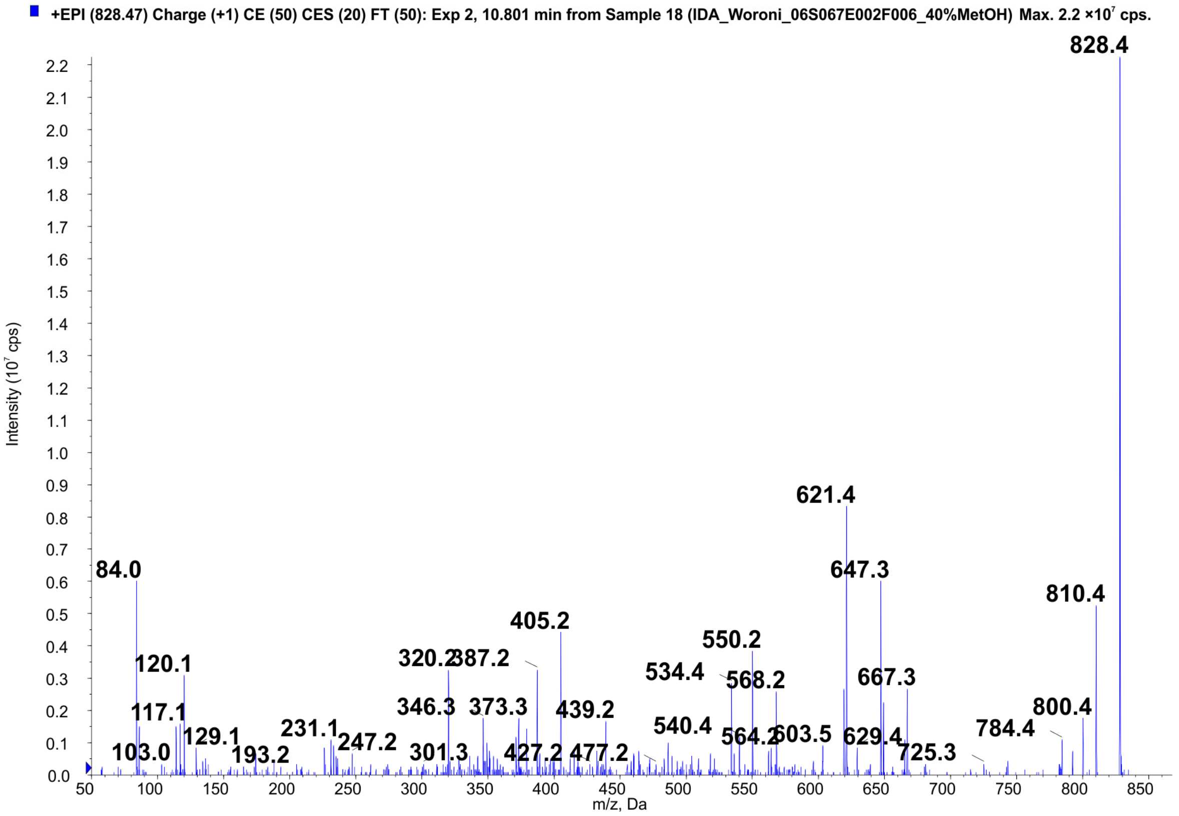

Figure S9. LC-MS/MS ion fragmentation spectrum of the suggested anabaenopeptin 827 and fragmented ions identified by their mass-to-charge ratios (*m*/*z*). The intensity of the ion on the *y*-axis is given as counted ions per second (cps) and the mass-to-charge ratio (*m*/*z*) on the *x*-axis. The suggested fragmented ions are at the *m*/*z* values: 828 [M + H]; 810 [M + H – H_2O]; 800 [M + H – CO]; 725 [M + H – *N*-MeAla – H_2O]; 647 [M + H – Tyr – H_2O]; 621 [M + H – (CO + Tyr)]; 603 [M + H – (CO + Tyr) – H_2O]; 534 [M + H – Tyr – *N*-MeAla – CO – H_2O]; 405 [M + H – Tyr – (Hph + Val)]; 387 [Lys + Val + Hph – H]; 373 [Phe + Lys + Val – H]; 320 [M + H – Tyr – (Val + Hph + *N*-MeAla)]; 247 [*N*-MeAla + Hph + H]; 231 [*N*-MeAla + Phe – H]; 120 Phe immonium ion; 84 Lys immonium ion.

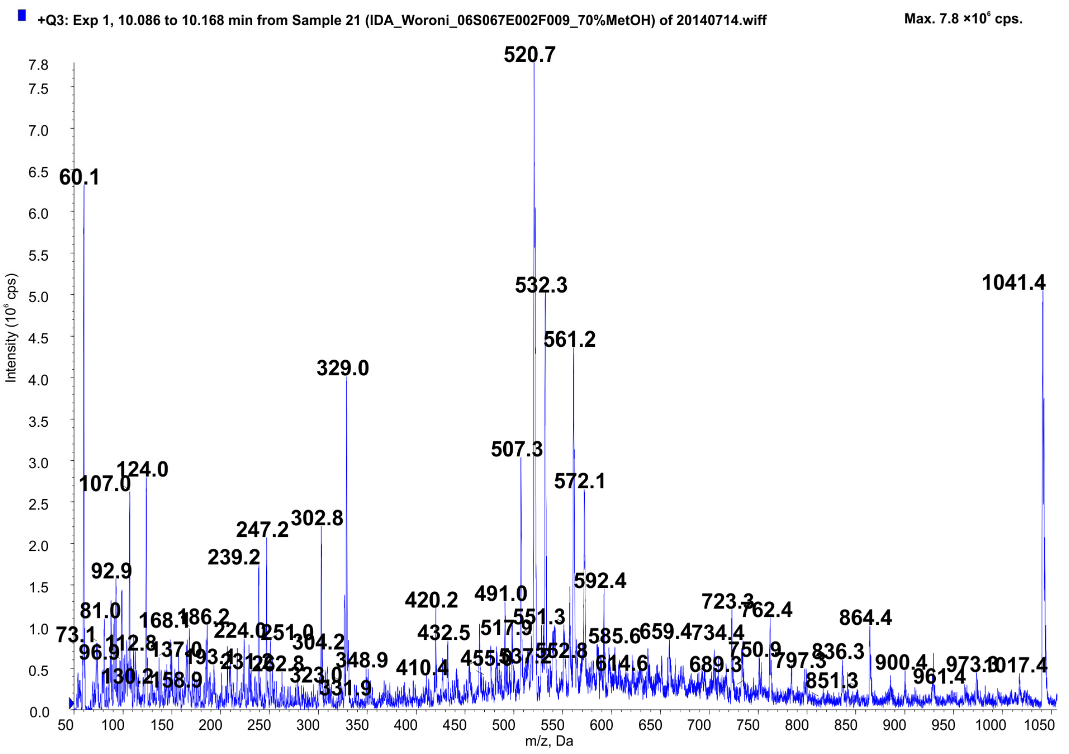

Figure S10. LC-MS/MS ion fragmentation spectrum of a suggested cyanopeptolin-like compound and fragmented ions identified by their mass-to-charge ratios (*m*/*z*). The intensity of the ion on the *y*-axis is given as counted ions per second (cps) and the mass-to-charge ratio (*m*/*z*) on the *x*-axis. The suggested fragmented ions are at the *m*/*z* values: 800 [the ring structure]; 521 [Lys + Ahp + Phe + MeTyr – H_2O – CO + H]; 420 [Ahp + Phe + MeTyr – H_2O + H].

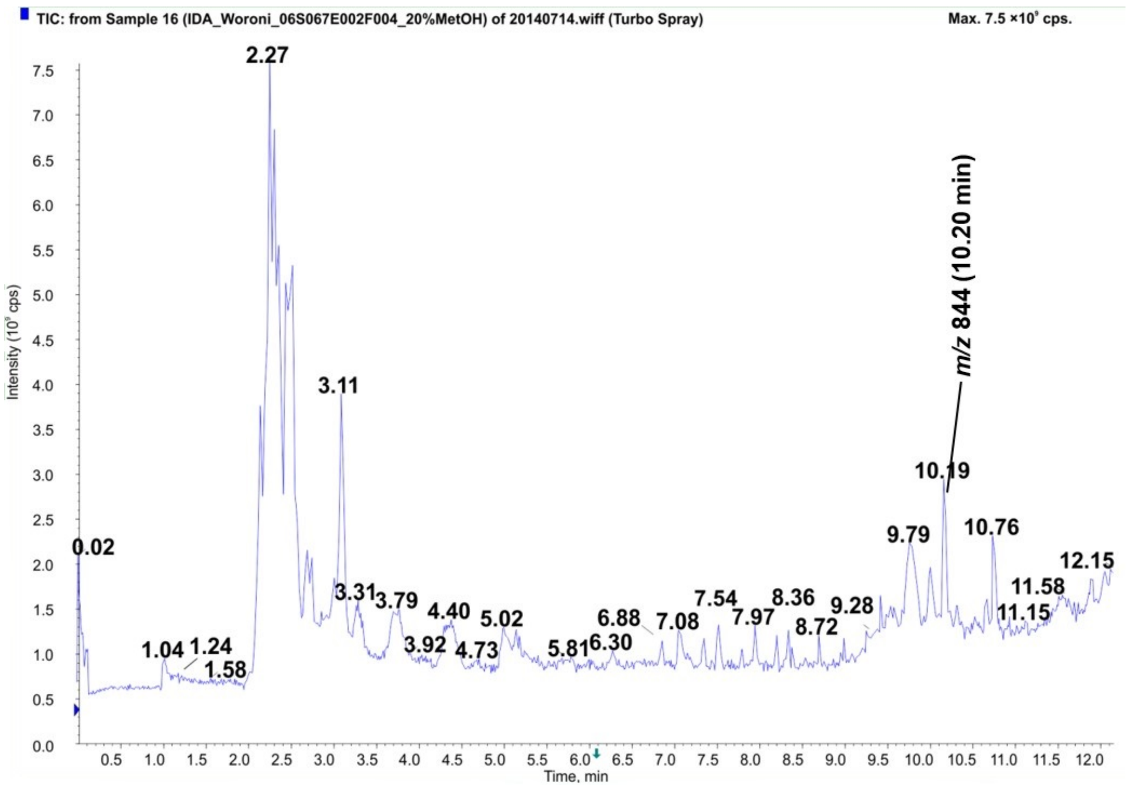

Figure S11. LC-MS/MS chromatogram of the 20% methanol fraction indicating the compound at *m*/*z* 844. The intensity of the ion on the *y*-axis is given as counted ions per second (cps) and the time (minutes) on the *x*-axis.

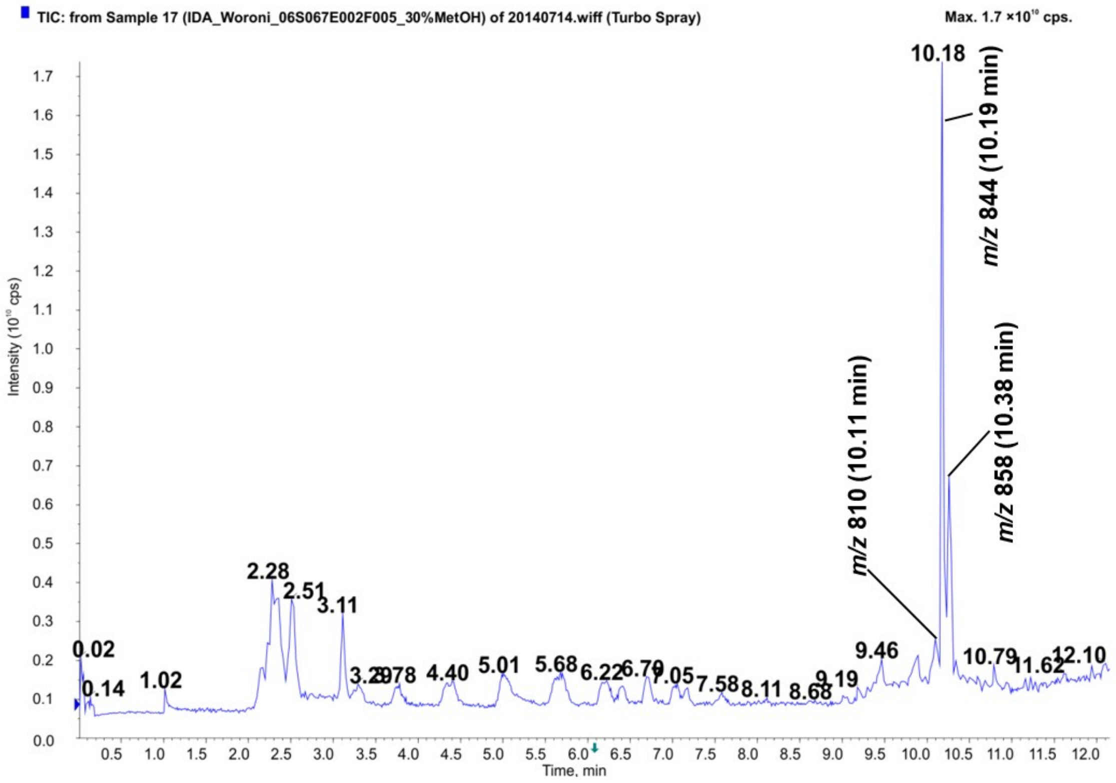

Figure S12. LC-MS/MS chromatogram of the 30% methanol fraction indicating the compounds at *m*/*z* 810, 844 and 858. The intensity of the ion on the *y*-axis is given as counted ions per second (cps) and the time (minutes) on the *x*-axis.

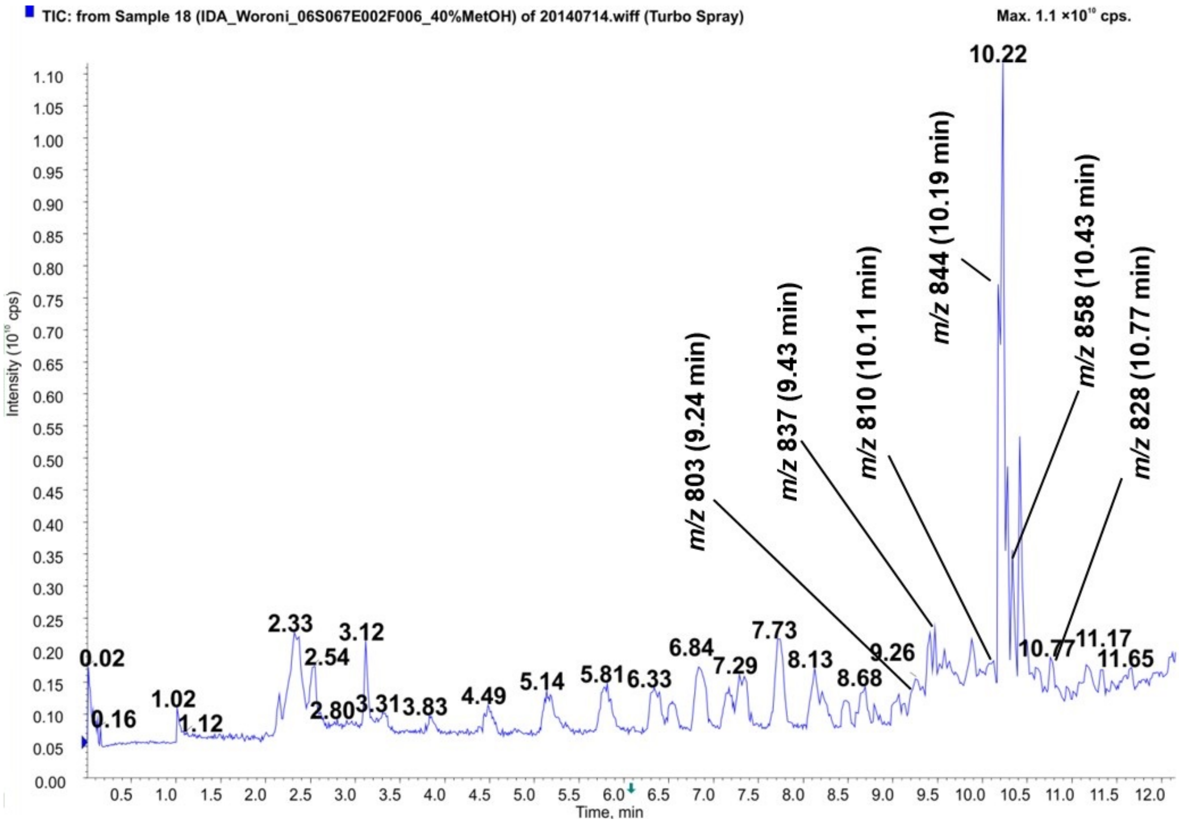

Figure S13. LC-MS/MS chromatogram of the 40% methanol fraction indicating the compounds at *m*/*z* 803, 810, 828, 837, 844 and 858. The intensity of the ion on the *y*-axis is given as counted ions per second (cps) and the time (minutes) on the *x*-axis.

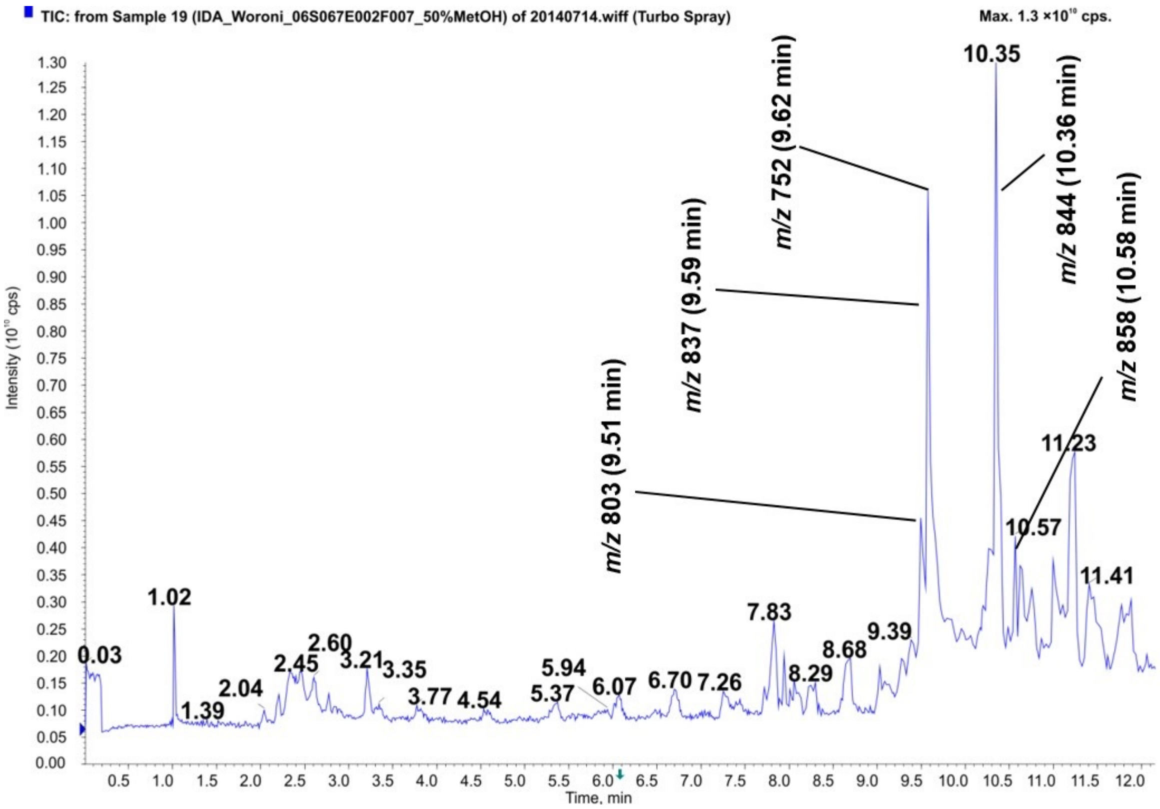

Figure S14. LC-MS/MS chromatogram of the 50% methanol fraction indicating the compounds at *m*/*z* 752, 803, 837, 844 and 858. The intensity of the ion on the *y*-axis is given as counted ions per second (cps) and the time (minutes) on the *x*-axis.

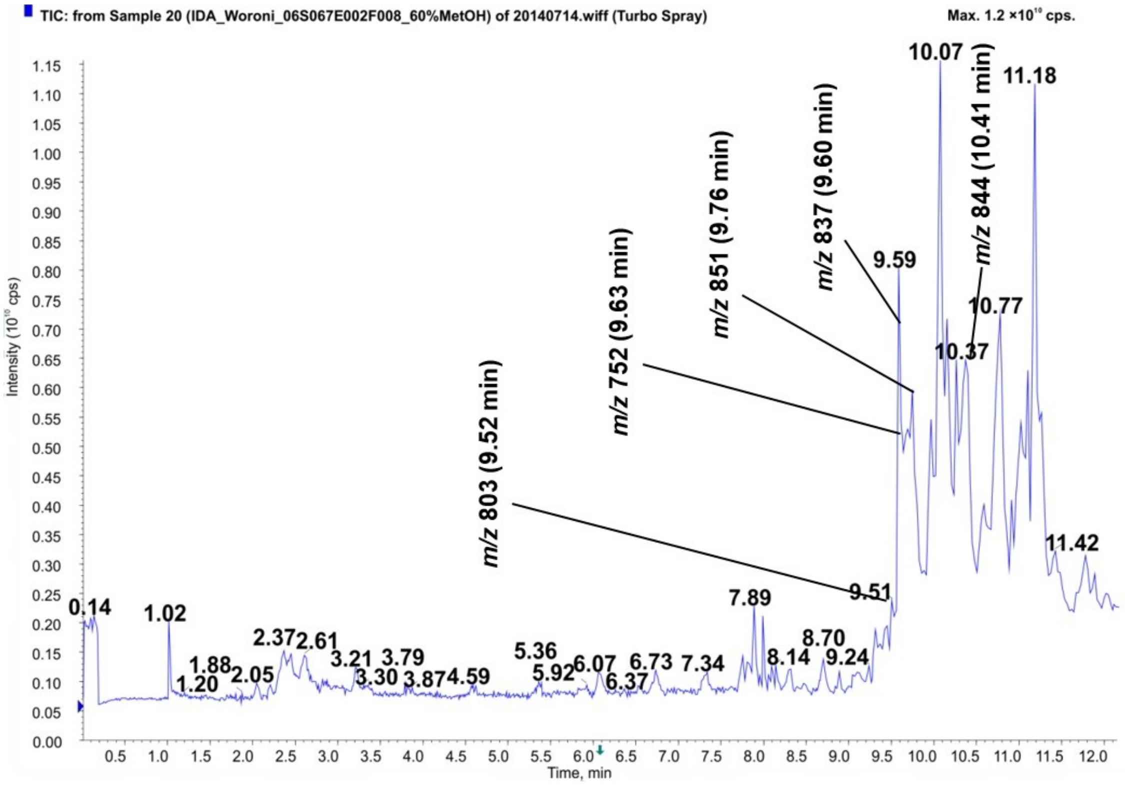

Figure S15. LC-MS/MS chromatogram of the 60% methanol fraction indicating the compounds at *m*/*z* 752, 803, 837, 844 and 851. The intensity of the ion on the *y*-axis is given as counted ions per second (cps) and the time (minutes) on the *x*-axis.

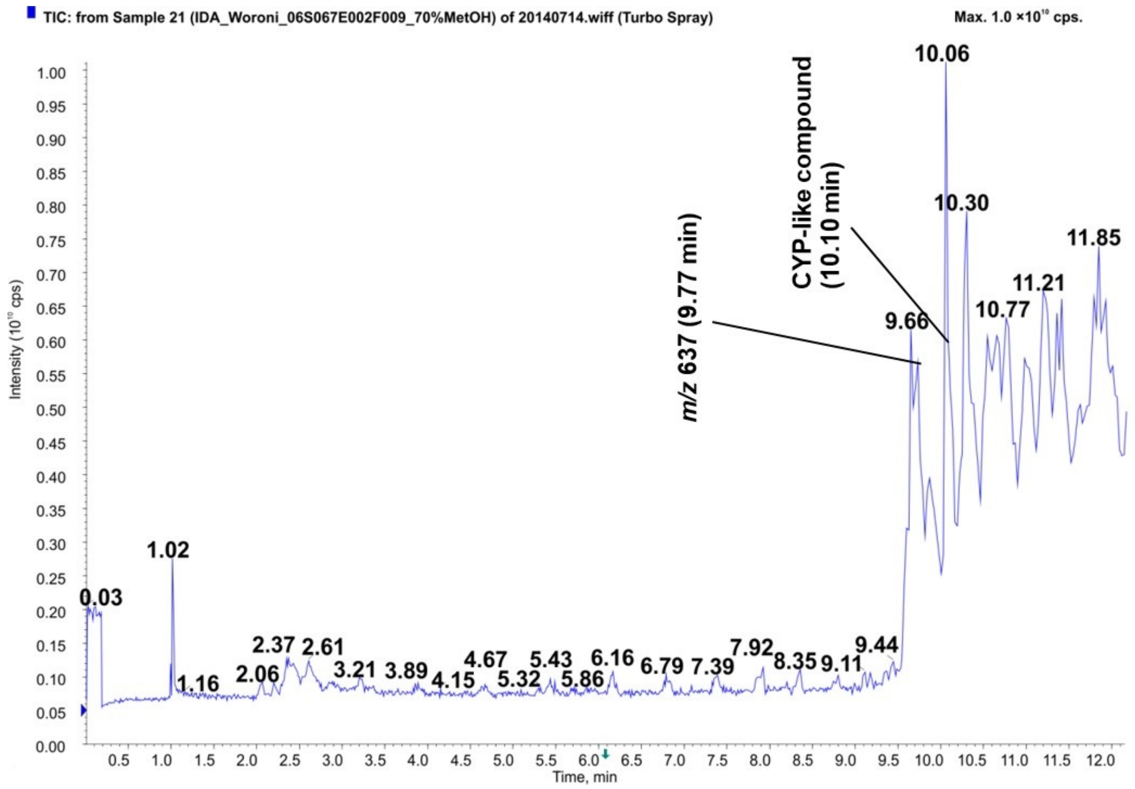

Figure S16. LC-MS/MS chromatogram of the 70% methanol fraction indicating the compound at *m*/*z* 637 and a cyanopeptolin-like (CYP-like). The intensity of the ion on the *y*-axis is given as counted ions per second (cps) and the time (minutes) on the *x*-axis.

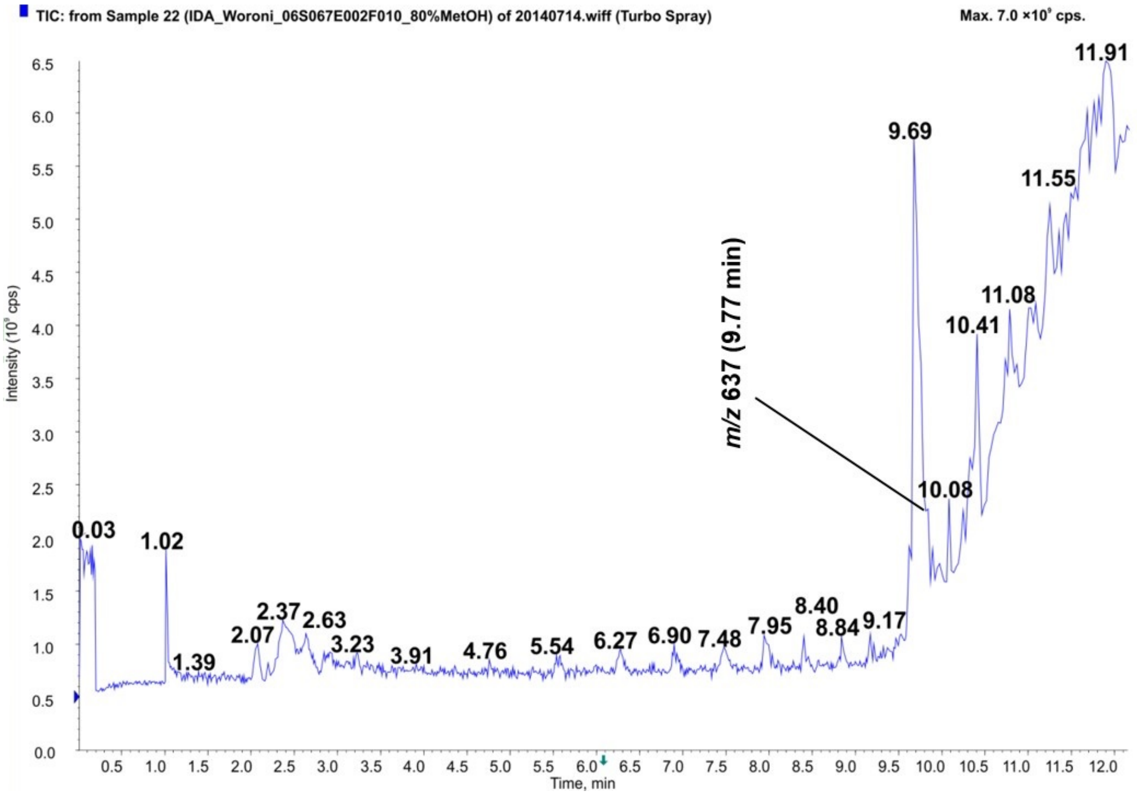

Figure S17. LC-MS/MS chromatogram of the 80% methanol fraction indicating the compound at *m*/*z* 637. The intensity of the ion on the *y*-axis is given as counted ions per second (cps) and the time (minutes) on the *x*-axis.

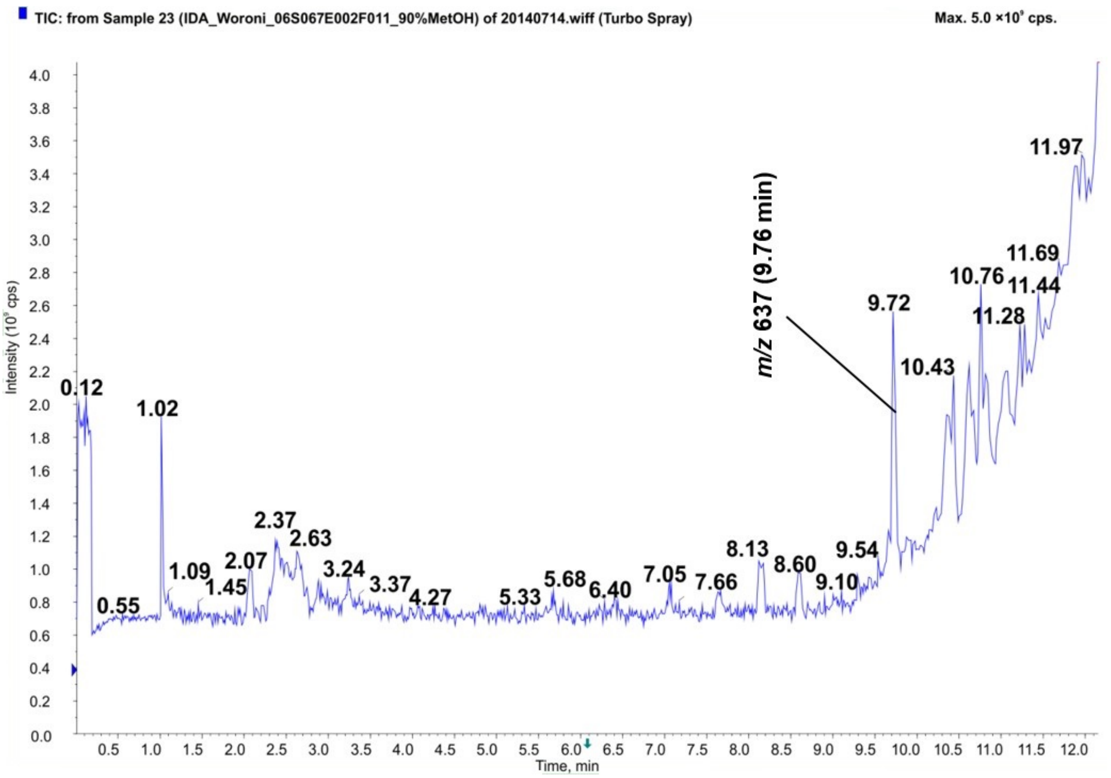

Figure S18. LC-MS/MS chromatogram of the 90% methanol fraction indicating the compound at *m*/*z* 637. The intensity of the ion on the *y*-axis is given as counted ions per second (cps) and the time (minutes) on the *x*-axis.

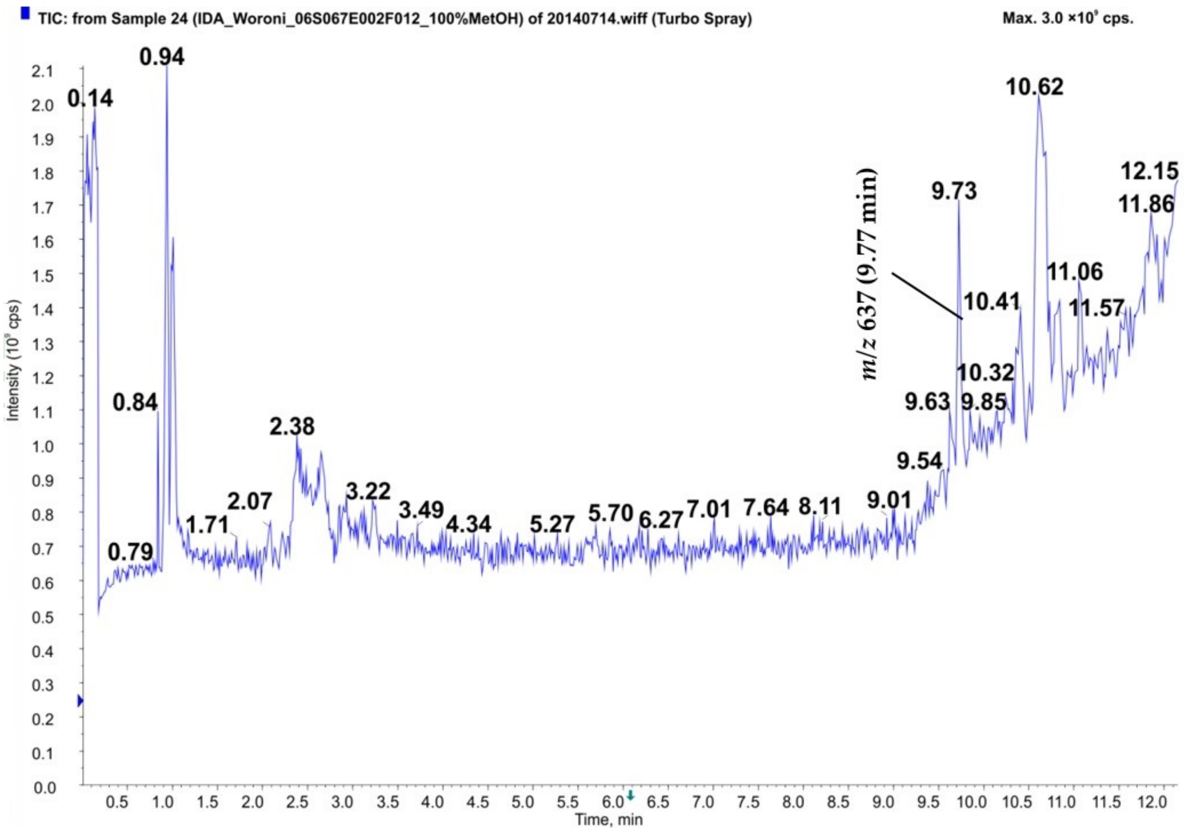

Figure S19. LC-MS/MS chromatogram of the 100% methanol fraction indicating the compound at *m*/*z* 637. The intensity of the ion on the *y*-axis is given as counted ions per second (cps) and the time (minutes) on the *x*-axis.
